# Association between oxidative balance score and 10-year atherosclerotic cardiovascular disease risk: results from the NHANES database

**DOI:** 10.3389/fnut.2024.1422946

**Published:** 2024-07-15

**Authors:** Dekui Jin, Tian Lv, Shiqin Chen, Yiqiao Chen, Chengying Zhang, Xiaoling Wang, Jie Li

**Affiliations:** ^1^Department of General Practice, The Third Medical Center of Chinese PLA General Hospital, Beijing, China; ^2^Department of Neurology, Zhuji Affiliated Hospital of Wenzhou Medical University, Shaoxing, Zhejiang, China; ^3^Yuhuan Second People’s Hospital, Yuhuan, China; ^4^Department of Neurology, Second People's Hospital of Yuhuan, Yuhan, China; ^5^Department of Neurology, Qingtian People’s Hospital, Qingtian, Lishui, China; ^6^Department of Neurology, Run Run Shaw Hospital, Zhejiang University, Hangzhou, China; ^7^Department of Neurology, Lishui People's Hospital, Lishui, China

**Keywords:** oxidative balance score, 10-year ASCVD risk, NHANES, dietary, lifestyle

## Abstract

**Introduction:**

The oxidative balance score (OBS) is a holistic measure that represents the overall equilibrium between prooxidants and antioxidants in one’s diet and lifestyle. Little research has been conducted on the correlation between OBS and 10-year atherosclerotic cardiovascular disease risk (ASCVD). Therefore, the objective of this investigation was to examine the potential correlation between OBS and 10-year risk.

**Methods:**

A total of 11,936 participants from the NHANES conducted between 2001 and 2016 were chosen for the study and their dietary and lifestyle factors were used to assess the OBS score. Logistic regression and restricted cubic splines (RCS) were employed in the cross-sectional study to evaluate the correlation between OBS and the 10-year ASCVD risk. The cohort study utilized Cox proportional hazards models and RCS to assess the correlation between OBS and all-causes and cardiovascular disease (CVD) mortality in individuals with high ASCVD risk.

**Results:**

The cross-sectional study found that the OBS (OR = 0.94, 95% CI = 0.93–0.98), as well as the dietary OBS (OR = 0.96, 95% CI = 0.92–0.96) and lifestyle OBS (OR = 0.74, 95% CI = 0.69–0.79), were inversely associated with the 10-year ASCVD risk. A significant linear relationship was observed between OBS, dietary OBS, lifestyle OBS, and the 10-year ASCVD risk. The cohort study found that the OBS was inversely associated with all-cause (aHRs = 0.97, 95% CI = 0.96–0.99) and CVD (aHRs = 0.95, 95% CI = 0.93–0.98) mortality in individuals with high ASCVD risk. A significant linear correlation was observed between OBS, dietary OBS, lifestyle OBS, and all-cause and CVD mortality in participants with high ASCVD risk.

**Conclusion:**

The findings indicate that OBS, OBS related to diet, and OBS related to lifestyle were significantly inversely correlated with the 10-year ASCVD risk. Adopting a healthy eating plan and making positive lifestyle choices that result in increased OBS levels can help lower the likelihood of all-cause and CVD mortality in individuals with high ASCVD risk.

## Background

Cardiovascular disease (CVD) is a condition affecting the heart and blood vessels and is recognized as the primary reason for global mortality (1). Hence, it is crucial to avert cardiovascular disease (CVD) and its related complexities while lessening the impact of the ailment and mortality rate. The accurate evaluation of CVD risk is crucial for formulating effective prevention and screening strategies. The recent most-utilized tool for evaluating the 10-year risk of atherosclerotic CVD (ASCVD), introduced by the American Heart Association (AHA) and the American College of Cardiology (ACC), is the 10-year ASCVD risk index (2).

It is widely recognized that detrimental habits, such as a lack of physical activity, an unhealthy eating pattern, smoking, and excessive alcohol consumption, have been linked to cardiovascular disease (CVD) occurrence and untimely fatalities (3, 4). Therefore, it is crucial to emphasize the potential for reducing CVD mortality by adopting healthier lifestyle choices. Maintaining a nutritious eating plan is a fundamental approach to proactively prevent cardiovascular diseases (5, 6). Observational research indicates that adopting a nutritious diet is associated with a decreased likelihood of experiencing cardiovascular events. Consequently, numerous individuals are urging for more robust public policies that encourage the consumption of healthy food options (7). Meanwhile, critics point out the lack of causal evidence linking a healthy diet with cardiovascular disease(CVD) prevention (8) and there are few studies looking at a healthy diet that addresses the primary prevention of CVD (9).

Various OBSs have been utilized in epidemiological research to consider both dietary and non-dietary lifestyle exposures (10). The OBS is a holistic measure that represents the overall equilibrium between prooxidants and antioxidants in one’s diet and lifestyle. In general, a higher OBS suggests that there are more antioxidants than prooxidants. Many studies have reported the negative associations between OBS and type 2 diabetes (11), osteoarthritis (12), cardiovascular disease (13), and cancers (14, 15). However, several studies have also documented the link between OBS and its constituents with various illnesses, including ASCVD mortality, and arrived at a contrasting outcome (16–19). This could be attributed to the intricate connections and associations among numerous prooxidant and antioxidant elements (10, 20), making it challenging to determine the individual impacts of these factors on disease susceptibility.

We consider that it is difficult to determine the roles played by exposure to dietary and non-dietary lifestyles and it is necessary to consider them as a complete whole. To summarize, there is insufficient evidence to determine the expected reduction in 10-year ASCVD risk resulting from established lifestyle and dietary interventions. Therefore, to gain a deeper comprehension of the impact of OBS, this study utilizes information from the NHANES 2001–2016, a comprehensive and representative survey of the American populace. It aims to explore the potential correlation between OBS and the risk of ASCVD over a span of 10 years. The results of our study could provide an important understanding of the involvement of OBS in the progression of ASCVD, thereby offering vital knowledge for the global prevention of ASCVD.

## Materials and methods

### Study design and participants

The data from the National Health and Nutrition Examination Survey data set of the United States (NHANES) were employed in this study. The Centers for Disease Control and Prevention (CDC) conducted a comprehensive cross-sectional study to assess the general health and nutritional well-being of the American population. This survey utilized a meticulously chosen representative sample of the United States populace. The NHANES dataset consists of five main parts, including information on demographics, diet, physical exams, lab results, and questionnaire answers. To collect pertinent data, the survey includes comprehensive interviews and extensive physical evaluations. Collecting data is essential for shaping public health policies and programs, assessing the effectiveness of health and nutrition initiatives, and monitoring the prevalence of different diseases and conditions. The National Center for Health Statistics Research Ethics Review Board duly approved all NHANES protocols, and all study participants provided informed consent prior to their involvement. Furthermore, all studies carried out strictly followed the applicable protocols and rules. To obtain extensive information about the collection of NHANES data, individuals who were interested could refer to the published materials accessible at https://www.cdc.gov/nchs/nhanes.htm.

**Figure 1:**
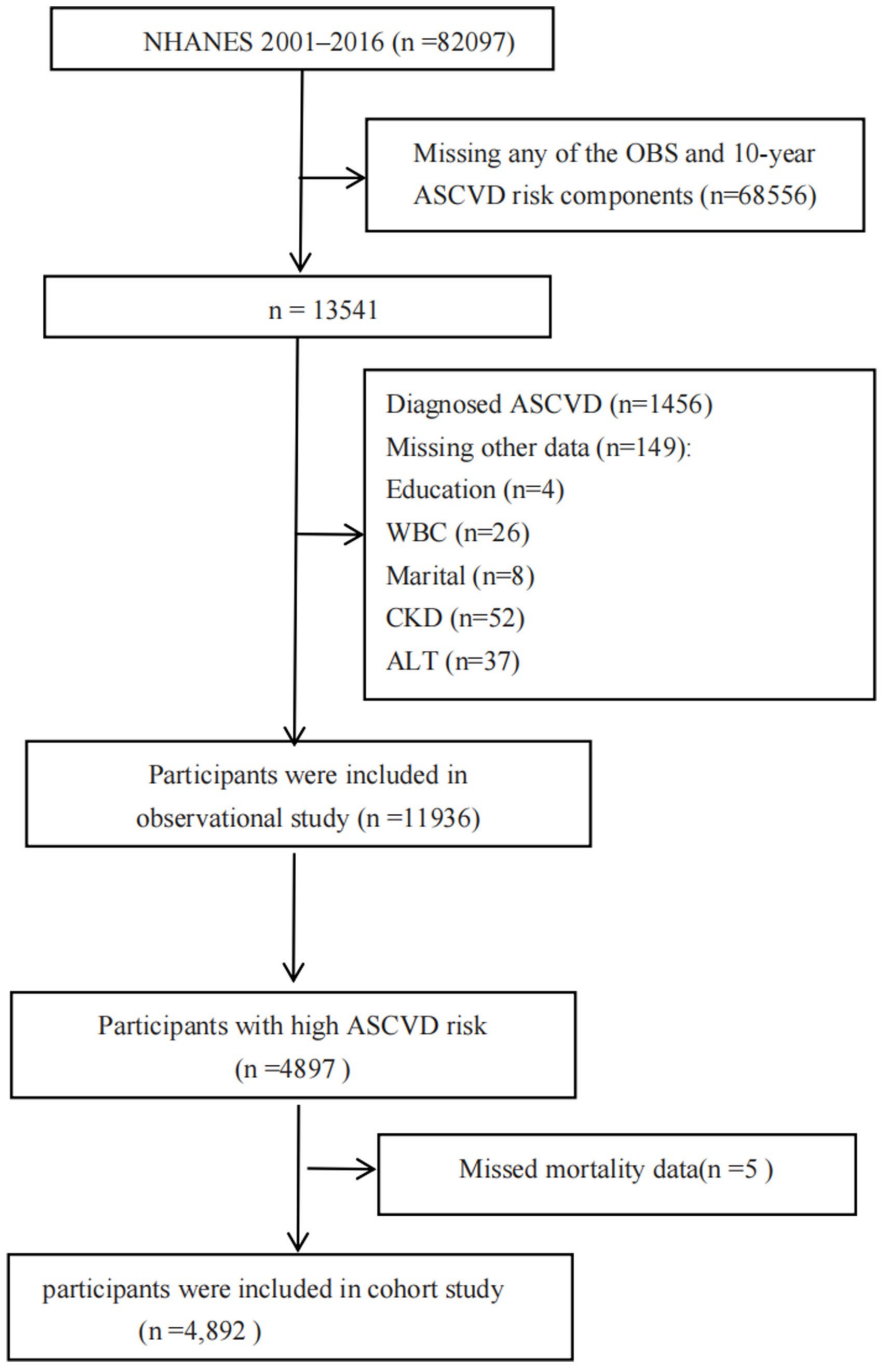
Flow chart of the study population.

In NHANES 2001–2016, a total of 82,097 individuals were initially registered. Exclusion criteria for individuals included: (1) loss of data for any of the OBS components (*n* = 9,034) or 10-year ASCVD risk (*n* = 59,522); (2) diagnosis of ASCVD in participants (*n* = 1,456); (3) missing other data such as education (*n* = 5), marital status (*n* = 8), white blood cell count (WBC) (*n* = 27), chronic kidney disease (CKD) (*n* = 65), alanine transaminase (ALT) (*n* = 38), and lymphocyte count (LYM) (*n* = 23). In the end, a total of 11,936 participants were included in the observational study (Figure 1). In the cohort study, we initially enrolled 4,897 individuals with high ASCVD risk. Five participants were excluded because missed mortality data. A total of 4,892 participants were included finally.

### Variable definition

The OBS, which is a holistic measure indicating the equilibrium between prooxidants and antioxidants in one’s diet and lifestyle, was determined by evaluating 16 nutrients and four lifestyle factors. This evaluation included five pro-oxidants and 15 antioxidants, using existing knowledge of the association between OBS and these factors (21). The nutrient intake data were obtained from the initial dietary review interview. As per the methodology employed by Zhang et al. (21). To determine OBS, alcohol intake was divided into three categories: heavy drinkers (15 g/day for females and 30 g/day for males), non-heavy drinkers (0–15 g/day for females and 0–30 g/day for males), and abstainers. Each group was assigned scores of 0, 1, and 2, respectively (21). Following this, the remaining elements were initially categorized into two sets according to gender and then further divided into three sets based on their tertile. In groups 1–3, pro-oxidants were assigned scores ranging from 2 to 0, while antioxidants were assigned scores ranging from 0 to 2. In general, a higher OBS suggests a prevalence of antioxidants compared to prooxidants (21).

The American College of Cardiology (ACC)/American Heart Association (AHA) guidelines for ASCVD 10-year risk assessment consider nine factors: race, sex, age, total cholesterol, HDL cholesterol, BP, diabetes, hypertension treatment, and smoking, we were able to categorize the study samples into two distinct groups. Individuals who have 10-year ASCVD risk <7.5% were categorized as having a “low ASCVD risk” and 2, those with 10-year ASCVD risk ≥7.5% were defined “high ASCVD risk” (22).

### Assessment of covariates

In this study, the covariates were certain factors previously shown or hypothesized to be associated with ASCVD or OBS, including demographic factors, blood examination, lifestyle, and concomitant disease. The demographic factors included the following: age, gender (male/female), ethnicity (black, white, other), level of education (less than high school, high school, college), Body Mass Index (BMI), and marital status. The blood test results included WBC, LYM, ALT, total cholesterol level (TC), high-density lipoprotein level (HDL), creatinine level/glomerular filtration rate (GFR), albumin level, hemoglobin level, levels of trace elements in the blood, dietary fiber level, carotene level, riboflavin level, niacin level, total folate level, cotinine level, and vitamin levels. Lifestyle includes of healthy eating index, alcohol use, smoking, total energy intake, and physical activity. Accompanying conditions included: diabetes mellitus (DM), hypertension, hyperlipidemia, and anemia.

### Statistical analyses

Our study considered the NHANES analytic guidelines, and considered complex sampling designs and sampling weights. Mobile examination centers (MECs) weights were utilized for all analyses. Continuous variables are presented as means and standard errors (SEs), while categorical variables are presented as proportions. The OBS were divided into four groups based on quartiles. Student’s *t*-test was used to compare continuous variables among the quartiles of the OBS groups, while chi-square test was used for categorical variables.

Three models were built to provide statistical inference. The first model solely consisted of OBS. Model 2 consisted of Model 1, sex, age, ethnicity, and educational background. The inclusion of Model 2 in Model 3 comprised creatinine, LYM, WBC, ALT, alcohol user, DM, hypertension, hyperlipidemia, anemia, and total energy intake.

A logistic regression analysis was conducted in the cross-sectional study to determine the adjusted odds ratio (aOR) and 95% CIs for the correlation between OBS and the risk of ASCVD over a period of 10 years. To flexibly model the relationship between OBS and 10-year ASCVD risk, we employed restricted cubic spline (RCS) regression.

The cohort study utilized Cox proportional hazards models to calculate adjusted hazard ratios (aHRs) and 95% CI, assessing the correlation between OBS and all-causes, CVD mortality in individuals with a high predicted 10-year ASCVD risk. The association of OBS with all-cause mortality and CVD mortality was modeled using Kaplan–Meier survival analysis in a flexible manner.

R Studio 4.2.0 was utilized for statistical analyses. A significance level of *p* < 0.05 was established for the statistical analysis.

## Results

### The cross-sectional study

#### Basic characteristics

This study utilized a NHANES dataset comprising 11,936 participants. The basic features of the 11,936 participants are displayed in Table 1. Among the total sample, 6,072 (49.146%) were male. OBS were categorized into quartiles: Q1 (3–15), Q2 (16–21), Q3 (22–26), and Q4 (27–37). Significant differences were observed between OBS quartiles and various variables, such as age, BMI, race, education, marital status, smoking, SBP, TC, HDL, dietary fiber, carotene, alpha-carotene, beta-carotene, riboflavin, niacin, total folate, vitamin B12, vitamin B6, vitamin C, vitamin E, calcium, magnesium, zinc, copper, total fat, iron, physical activity, cotinine, anemia, CKD, hypertension, hyperlipidemia, DM, 10-year ASCVD risk, and high ASCVD risk (all *p* < 0.0001). In addition, there were significant differences in hemoglobin, physical activity, and alcohol across the OBS quartiles (*p* < 0.005). It was important to note that the participants with high ASCVD risk also showed a significant trend from the lower OBS quartiles to the high OBS quartiles (*p* value <0.001).

**Table 1:** Baseline characteristics of study participants based on the OSB quartiles.

Variable	Total	Q1	Q2	Q3	Q4	*p*-value
Age (year)	54.765 (0.149)	54.689 (0.235)	55.003 (0.235)	55.005 (0.245)	54.284 (0.280)	0.145
Sex						0.054
Female	5,864 (50.854)	1,355 (47.834)	1,526 (50.597)	1,700 (51.372)	1,283 (53.253)	
Male	6,072 (49.146)	1,683 (52.166)	1,570 (49.403)	1,660 (48.628)	1,159 (46.747)	
BMI (kg/m^2^)	28.645 (0.095)	29.476 (0.147)	29.211 (0.149)	28.586 (0.154)	27.358 (0.165)	<0.0001^*^
Race						<0.0001*
White	6,075 (77.909)	1,326 (71.562)	1,542 (77.016)	1,821 (80.199)	1,386 (81.830)	
Black	2,312 (8.344)	883 (14.411)	624 (8.973)	514 (6.391)	291 (4.539)	
Other	3,549 (13.747)	829 (14.027)	930 (14.011)	1,025 (13.410)	765 (13.631)	
Education						<0.0001*
<high	2,471 (11.609)	876 (18.049)	709 (13.047)	570 (9.640)	316 (6.626)	
High	2,717 (22.363)	828 (28.695)	731 (25.728)	743 (20.814)	415 (14.917)	
College	6,748 (66.027)	1,334 (53.256)	1,656 (61.224)	2,047 (69.546)	1,711 (78.457)	
Marital						<0.0001*
Yes	7,559 (67.651)	1,761 (61.122)	1,938 (66.371)	2,210 (69.643)	1,650 (72.522)	
No	4,377 (32.349)	1,277 (38.878)	1,158 (33.629)	1,150 (30.357)	792 (27.478)	
Alcohol.user						<0.0001*
Never	1,458 (9.639)	374 (11.045)	413 (10.829)	400 (9.085)	271 (7.779)	
Former	2,345 (16.430)	718 (20.246)	598 (15.727)	595 (15.032)	434 (15.413)	
Mild	4,524 (41.547)	953 (32.427)	1,144 (39.335)	1,335 (44.014)	1,092 (49.189)	
Moderate	1,821 (17.440)	470 (17.247)	457 (16.704)	532 (18.258)	362 (17.377)	
Heavy	1,788 (14.944)	523 (19.035)	484 (17.405)	498 (13.611)	283 (10.243)	
Alcohol (g/day)	10.513 (0.358)	10.330 (0.672)	11.115 (0.751)	11.185 (0.563)	9.208 (0.581)	0.057
Smoke						<0.0001^*^
Yes	2,203 (17.426)	886 (30.661)	590 (19.126)	525 (14.084)	202 (7.635)	
No	9,733 (82.574)	2,152 (69.339)	2,506 (80.874)	2,835 (85.916)	2,240 (92.365)	
Physical activity (MET-minute/week)	2876.530 (68.968)	2623.927 (127.134)	2846.104 (121.182)	2947.550 (108.621)	3052.215 (114.543)	0.034^*^
SBP	124.378 (0.267)	126.592 (0.487)	125.737 (0.449)	123.882 (0.405)	121.524 (0.435)	<0.0001^*^
Total energy intake, Mean (S.E)	2142.145 (11.832)	1574.933 (17.543)	1972.425 (17.782)	2291.644 (20.394)	2656.452 (31.031)	<0.0001^*^
TC	206.450 (0.685)	208.433 (1.289)	206.546 (1.051)	206.130 (1.126)	204.928 (1.173)	0.203
HDL	55.115 (0.260)	53.522 (0.487)	53.739 (0.468)	55.680 (0.455)	57.329 (0.534)	<0.0001^*^
Alt	25.951 (0.216)	26.124 (0.459)	26.660 (0.565)	25.617 (0.286)	25.456 (0.466)	0.317
Blood urea nitrogen (mg/dL)	13.771 (0.084)	13.001 (0.140)	13.696 (0.120)	13.900 (0.117)	14.398 (0.142)	<0.0001^*^
Creatinine (mg/dL)	0.895 (0.003)	0.924 (0.008)	0.897 (0.005)	0.889 (0.005)	0.874 (0.005)	<0.0001^*^
Albumin (g/L)	42.985 (0.051)	42.569 (0.097)	42.918 (0.083)	43.077 (0.074)	43.325 (0.075)	<0.0001^*^
Wbc_1000cells (/μL)	7.043 (0.030)	7.363 (0.062)	7.172 (0.053)	7.015 (0.059)	6.648 (0.053)	<0.0001^*^
Lym	30.006 (0.104)	30.181 (0.190)	29.917 (0.189)	29.759 (0.194)	30.245 (0.186)	0.164
Neu	58.433 (0.111)	58.438 (0.222)	58.448 (0.227)	58.664 (0.211)	58.127 (0.221)	0.394
Hemoglobin (g/dL)	14.394 (0.032)	14.469 (0.046)	14.455 (0.046)	14.348 (0.046)	14.320 (0.041)	0.007^*^
Healthy eating index	53.210 (0.253)	46.330 (0.339)	51.057 (0.304)	54.584 (0.361)	60.096 (0.476)	<0.0001^*^
Dietary fiber (g/day)	17.807 (0.145)	9.876 (0.109)	14.562 (0.128)	19.245 (0.174)	26.735 (0.298)	<0.0001^*^
Carotene (RE/day)	238.507 (6.335)	103.602 (4.937)	179.929 (6.857)	243.795 (7.380)	417.803 (22.479)	<0.0001^*^
Alpha carotene (mcg/day)	495.118 (21.045)	495.118 (21.045)	495.118 (21.045)	495.118 (21.045)	495.118 (21.045)	<0.0001^*^
Beta carotene (mcg/day)	2614.526 (67.059)	1133.500 (53.827)	1968.792 (73.378)	2680.789 (80.458)	4575.477 (233.678)	<0.0001^*^
Riboflavin (mg/day)	2.269 (0.016)	1.445 (0.015)	1.973 (0.018)	2.450 (0.023)	3.112 (0.033)	<0.0001^*^
Niacin (mg/day)	25.045 (0.158)	16.327 (0.173)	22.115 (0.252)	27.193 (0.230)	33.477 (0.380)	<0.0001^*^
Total folate (mcg/day)	417.050 (3.336)	242.641 (2.103)	339.682 (3.286)	451.642 (3.933)	616.029 (7.580)	<0.0001^*^
Vitamin B12 (mcg/day)	5.399 (0.088)	2.917 (0.064)	4.365 (0.089)	6.137 (0.213)	7.853 (0.162)	<0.0001^*^
Vitamin B6 (mg/day)	2.073 (0.016)	1.201 (0.014)	1.735 (0.023)	2.257 (0.022)	3.002 (0.034)	<0.0001^*^
Vitamin C (mg/day)	87.605 (1.243)	45.663 (1.266)	67.817 (1.487)	93.269 (2.107)	140.012 (2.783)	<0.0001^*^
Vitamin E (ATE) (mg/day)	8.541 (0.094)	4.646 (0.062)	6.841 (0.076)	9.272 (0.107)	13.008 (0.217)	<0.0001^*^
Calcium (mg/day)	943.926 (7.114)	561.495 (6.678)	802.827 (8.451)	1020.033 (9.917)	1349.674 (15.124)	<0.0001^*^
Magnesium (mg/day)	312.993 (2.121)	187.249 (1.490)	263.576 (1.754)	339.453 (2.254)	447.816 (3.871)	<0.0001^*^
Zinc (mg/day)	11.970 (0.093)	7.363 (0.093)	10.158 (0.129)	13.144 (0.179)	16.656 (0.199)	<0.0001^*^
Copper (mg/day)	1.370 (0.013)	0.813 (0.008)	1.131 (0.010)	1.516 (0.030)	1.953 (0.025)	<0.0001^*^
Total fat (g/day)	81.767 (0.529)	58.170 (0.658)	75.980 (0.761)	88.532 (0.908)	101.136 (1.277)	<0.0001^*^
Iron (mg/day)	15.497 (0.111)	9.657 (0.106)	13.070 (0.116)	16.761 (0.156)	21.854 (0.263)	<0.0001^*^
Selenium (mcg)	112.991 (0.640)	75.700 (0.779)	100.885 (0.966)	122.215 (1.152)	148.561 (1.737)	<0.0001^*^
Cotinine (ng/mL)	53.587 (2.189)	96.752 (4.360)	60.517 (3.941)	40.254 (2.780)	23.224 (2.565)	<0.0001^*^
Anemia						0.052
Non-Anemia	11,073 (95.011)	2,770 (93.625)	2,877 (95.461)	3,131 (94.798)	2,295 (96.072)	
Mild	649 (3.655)	201 (4.344)	166 (3.327)	169 (3.884)	113 (3.082)	
Moderate	202 (1.264)	65 (1.908)	49 (1.129)	56 (1.270)	32 (0.808)	
Severe	12 (0.070)	2 (0.123)	4 (0.083)	4 (0.047)	2 (0.037)	
CKD						<0.0001^*^
Yes	1,761 (12.391)	585 (16.394)	458 (13.437)	446 (11.433)	272 (8.803)	
No	10,175 (87.609)	2,453 (83.606)	2,638 (86.563)	2,914 (88.567)	2,170 (91.197)	
Hypertension						<0.0001^*^
Yes	5,619 (42.957)	1,613 (47.327)	1,499 (46.680)	1,521 (42.598)	986 (35.447)	
No	6,317 (57.043)	1,425 (52.673)	1,597 (53.320)	1,839 (57.402)	1,456 (64.553)	
Hyperlipidemia						<0.0001^*^
Yes	9,308 (78.567)	2,422 (80.606)	2,473 (81.662)	2,613 (77.810)	1,800 (74.361)	
No	9,308 (78.567)	2,422 (80.606)	2,473 (81.662)	2,613 (77.810)	1,800 (74.361)	
DM						<0.0001^*^
Yes	2,146 (13.133)	645 (15.302)	584 (14.848)	598 (13.336)	319 (9.070)	
No	9,790 (86.867)	2,393 (84.698)	2,512 (85.152)	2,762 (86.664)	2,123 (90.930)	
10-year ASCVD risk	0.080 (0.001)	0.094 (0.002)	0.085 (0.003)	0.078 (0.002)	0.062 (0.002)	<0.0001
High predicted 10-year ASCVD risk						<0.0001^*^
Yes	4,897 (33.204)	1,489 (39.133)	1,334 (37.014)	1,287 (32.205)	787 (24.970)	
No	7,039 (66.796)	1,549 (60.867)	1,762 (62.986)	2,073 (67.795)	1,655 (75.030)	

Mean ± SEs for continuous variables: *p* value was calculated by weighted Student’s *t-*test. Number (%) for categorical variables: *p* value was calculated by weighted chi-square test. BMI, Body mass index; Edu, Education; LYM, Lymphocyte ratio; WBC, Leucocyte count; Neu, Neutrophile granulocyte; 10-year ASCVD risk, 10-year atherosclerotic cardiovascular disease risk; DM, Diabetes mellitus; CKD, Chronic kidney disease; TC, Cholesterin; HDL, High density lipoprotein; SBP, Systolic pressure; High predicted 10-year risk, 10-year ASCVD risk ≥ 7.5%.

#### Association between OBS and high ASCVD risk

After making adjustments for different models, Table 2 demonstrates the correlation between OBS and high ASCVD risk. The OBS was analyzed both as a continuous and a categorized variable. After making adjustments to different models in the ongoing model, the outcomes for Model 1 indicated aOR = 0.97 [95% CI (0.96–0.97), *p* < 0.0001]. Similarly, Model 2 showed aORs = 0.94 [95% CI (0.93–0.95), *p* < 0.0001]. In addition, Model 3 exhibited aOR = 0.94 [95% CI of (0.92–0.96), *p* < 0.0001]. After adjusting Model 3 in the OBS categorized model, the aOR and their corresponding 95% confidence intervals (CI) for different OBS categories (3–15, 16–21, 22–26, and 27–37) were as follows: 1.00 (reference), 0.79 (0.61, 1.03), (*p* = 0.08), 0.56 (0.41, 0.77), (*p* < 0.0001), and 0.39 (0.27, 0.56), (*p* < 0.0001) for high ASCVD risk.

**Table 2:** Multivariable logistic regression analyses demonstrating associations of OBS and 10-year ASCVD risk.

	Multivariable adjusted (OR, 95% CI)^*^					
	Model 1		Model 2		Model 3	
OBS	95%CI	*p*-value	95%CI	*p*-value	95%CI	*p*-value
Q1	ref		ref		ref	
Q2	0.91 (0.79,1.05)	0.21	0.83 (0.66, 1.05)	0.11	0.79 (0.61, 1.03)	0.08
Q3	0.74 (0.63,0.86)	<0.001	0.57 (0.46, 0.71)	<0.0001	0.56 (0.41, 0.77)	<0.0001
Q4	0.52 (0.45,0.60)	<0.0001	0.34 (0.27, 0.45)	<0.0001	0.39 (0.27, 0.56)	<0.0001
*p* trend		<0.0001		<0.0001		<0.0001
OBS	0.97 (0.96,0.97)	<0.0001	0.94 (0.93, 0.95)	<0.0001	0.94 (0.92, 0.96)	<0.0001

Model 1 comprised OBS only. Model 2 included Model 1, sex, and age, race and education. Model 3 included Model 2, creatinine, lymphocyte ratio (LYM), leucocyte count (WBC), glutamic-pyruvic transaminase (ALT), alcohol user, diabetes mellitus (DM), hypertension, hyperlipidemia, anemia, and total energy intake. ^*^p < 0.05.

Supplementary Table S2 showed the associations of OBS with study outcomes in age, sex, GFR, hyperlipidemia, and hypertension subjects. In model 3 adjusted for age, sex, GFR, hyperlipidemia, and hypertension, there was a significant association between OBS and high ASCVD risk. Subgroup analyses showed results with no significant interaction (All *p* interaction >0.05).

#### Associations of dietary OBS, lifestyle OBS with high ASCVD risk

Supplementary Table S1 displays the outcomes of a multiple logistic regression analysis that assesses the correlation between dietary OBS, lifestyle OBS, and high ASCVD risk. After adjusting for Model 3, there was a significant negative association between high ASCVD risk and dietary OBS, with a statistically significant continuous OR of 0.96 (0.93, 0.98) and a categorized OR of 0.49 (0.33, 0.72), both with *p* < 0.0001. After adjusting for Model 3, there was a significant negative association between high ASCVD risk and lifestyle OBS. The association remained statistically significant with a continuous aOR of 0.74 (0.69, 0.79), *p* < 0.0001, and categorized OR of 0.34 (0.26, 0.45), *p* < 0.001.

Supplementary Table S2 showed the associations of dietary OBS, lifestyle OBS with study outcomes in age, sex, GFR, hyperlipidemia, and hypertension subjects.

In model 3, adjusted for sex, GFR, and hypertension, there was a significant association between dietary OBS and high ASCVD risk. In the younger (<60), one-unit increasement in dietary OBS, high ASCVD risk decreased by 4.9%. In the hyperlipidemia participants, one-unit increasement in dietary OBS, high 10-year ASCVD risk decreased by 4.5%. Subgroup analyses showed results with no significant interaction (All *p* interaction >0.05).

In model 3, adjusted for sex and hypertension, there was a significant association between lifestyle OBS and high ASCVD risk. In the younger (<60), one-unit increasement in lifestyle OBS, high ASCVD risk decreased by 16.4%. In the hyperlipidemia participants, one-unit increasement in lifestyle OBS, high 10-year ASCVD risk decreased by 27.5%. In the GFR <60 mL/min/1.73 m^2^ group, there was a no association between lifestyle OBS and high ASCVD risk. It was interesting to note that there was a significant association between lifestyle OBS and high 10-year ASCVD risk among those with age, GFR, and hyperlipidemia (*p* for interaction >0.05).

#### The high ASCVD risk is associated linearly with OBS, dietary OBS, and lifestyle OBS

**Figure 2:**
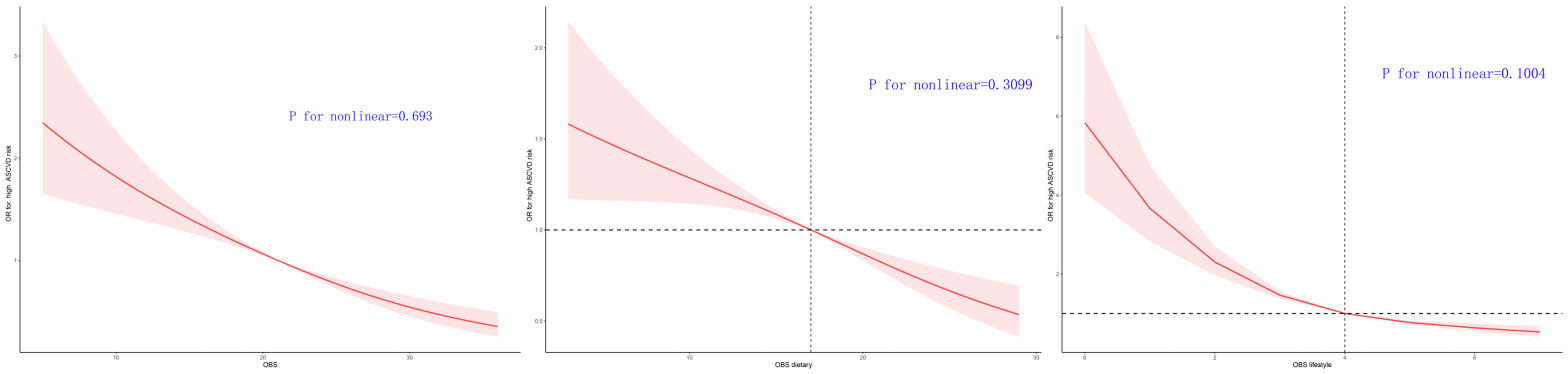
Multivariable-adjusted ORs for 10-year ASCVD risk by OBS, dietary OBS, and lifestyle OBS.

Following the adjustment for Model 3, a linear correlation was detected among OBS, dietary OBS, lifestyle OBS, and the high ASCVD risk (Figure 2). According to RCS models, there is a consistent decline in the aOR for the high ASCVD risk with an increase in OBS, dietary OBS, and lifestyle OBS (Figure 2).

In both the male and female groups, there was a noticeable correlation between OBS, dietary OBS, and the high ASCVD risk, as depicted in Supplementary Figures S1, S2. In the female group, a noteworthy non-linear association was found between lifestyle OBS and the high ASCVD risk (*p* value for nonlinearity = 0.0017, as shown in Supplementary Figure S3).

### Cohort study basic characteristics

#### Basic characteristics

In the cohort study, a total of 4,892 participants with high-risk 10-years ASCVD were included (Supplementary Table S3). The average length of follow-up was 115.0 months, with a range of 72.0–153.0 months (IQR). Out of the entire sample, 3,850 individuals (78.70%) were males, while there were 1,053 instances (19.5%) of recorded mortality. OBSs were categorized into quartiles: Q1 (3–15), Q2 (16–21), Q3 (22–26), and Q4 (27–37). It was important to note that the mortality also showed a significant trend from the lower OBS quartiles to the high OBS quartiles (*p* value <0.001) (Supplementary Table S3).

#### The connections between OBS and all-causes, CVD mortality in individuals with high ASCVD risk

The correlation between OBS and all-causes, CVD mortality in individuals with high ASCVD risk is depicted in Table 3. After adjusting various models in the OBS continuous model, the results showed that for Model 1, the aHRs = 0.98 (0.97–0.99), *p* value < 0.0001 (Table 3). Similarly, for Model 2, the aHRs = 0.97 (0.96–0.98), *p* < 0.0001. In addition, for Model 3, the aHRs = 0.97 (0.96–0.99), *p* < 0.0001 for all-cause mortality. For Model 1, the aHRs = 0.97 (0.95–0.99), *p* < 0.0001. Similarly, for Model 2, the aHRs = 0.95 (0.93–0.97), *p* < 0.0001. Furthermore, for Model 3, the aHRs were 0.95 (0.93–0.98) *p* < 0.002 for CVD mortality.

**Table 3:** Multivariable Cox regression analyses demonstrating associations of OBS and all-cause mortality, CVD mortality.

	Multivariable adjusted (HR, 95% CI)^*^					
OBS	Model 1		Model 2		Model 3	
	95%CI	*p*-value	95%CI	*p*-value	95%CI	*p*-value
All-cause mortality						
Q1	ref		ref		ref	
Q2	0.85 (0.68,1.07)	0.17	0.82 (0.65,1.03)	0.08	0.86 (0.67,1.10)	0.23
Q3	0.87 (0.70,1.07)	0.18	0.80 (0.65,0.99)	0.04	0.89 (0.69,1.14)	0.34
Q4	0.65 (0.50,0.84)	<0.001	0.57 (0.44,0.73)	<0.0001	0.66 (0.49,0.87)	0.004
*p* trend		0.002		<0.0001		0.01
OBS	0.98 (0.97,0.99)	<0.0001	0.97 (0.96,0.98)	<0.0001	0.97 (0.96,0.99)	<0.0001
CVD mortality						
Q1	ref		ref		ref	
Q2	0.71 (0.50,1.00)	0.05	0.66 (0.47,0.92)	0.01	0.66 (0.45,0.96)	0.03
Q3	0.62 (0.44,0.87)	0.01	0.54 (0.39,0.74)	<0.001	0.56 (0.36,0.85)	0.01
Q4	0.53 (0.33,0.84)	0.01	0.43 (0.27,0.69)	<0.001	0.45 (0.25,0.80)	0.01
*p* trend		0.004		<0.0001		0.01
OBS	0.97 (0.95,0.99)	<0.001	0.95 (0.93,0.97)	<0.0001	0.95 (0.93,0.98)	0.002

Model 1 comprised OBS only. Model 2 included Model 1, sex, and age, race and education. Model 3 included Model 2, creatinine, lymphocyte ratio (LYM), leucocyte count (WBC), glutamic-pyruvic transaminase (ALT), alcohol user, diabetes mellitus (DM), hypertension, hyperlipidemia, anemia, and total energy intake. ^*^p < 0.05.

In the OBS categorized model, after adjustment Model 3, the aHRs and corresponding to the 95% CI for the different OBS categories (Q1, Q2, Q3, and Q4) had the following values: 1.00 (reference), 0.86 (0.67, 1.10) (*p* = 0.23), 0.89 (0.69, 1.14) (*p* = 0.34), 0.66 (0.49, 0.87) (*p* = 0.004) for all-cause mortality (*p* trend = 0.01); and 1.00 (reference), 0.66 (0.45, 0.96) (*p* = 0.03), 0.56 (0.36, 0.85) (*p* = 0.01), 0.45 (0.25, 0.80) (*p* = 0.01) for CVD mortality (*p* trend = 0.01).

#### Examining the connection between dietary OBS, lifestyle OBS, and the risk of all-cause and CVD mortality in individuals with a highly predicted ASCVD risk over a 10-year period

Supplementary Table S4 displays the outcomes of cox regression analysis, which assesses the correlation between dietary OBS, lifestyle OBS, and the risk of ASCVD over a period of 10 years. After adjusting for Model 3, there was a significant negative association between dietary OBS and both all-cause mortality [HR = 0.98 (0.96, 0.99), *p* = 0.002] and CVD mortality [HR = 0.95 (0.93, 0.98), *p* = 0.002], and a significant negative association between lifestyle OBS and both all-cause mortality [HR = 0.94 (0.89, 0.99), *p* = 0.01] and CVD mortality [HR = 0.90 (0.82, 0.98), *p* = 0.02] (Supplementary Table S4).

#### The all-cause mortality and CVD mortality is associated linearly with OBS, dietary OBS, and lifestyle OBS

**Figure 3:**
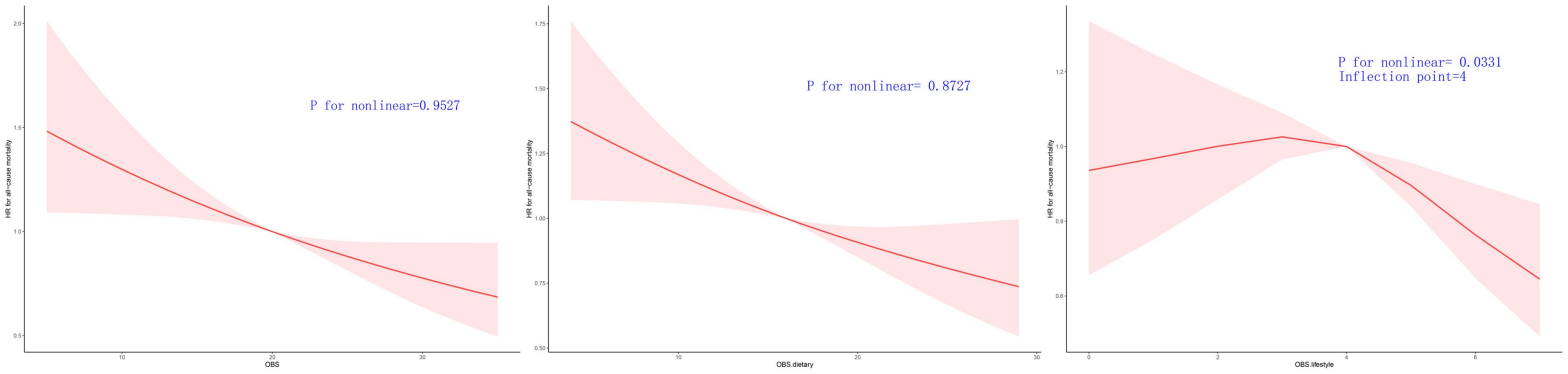
Multivariable-adjusted HRs for all cause mortality by OBS, diatery OBS, and lifestyle OBS.

**Figure 4:**
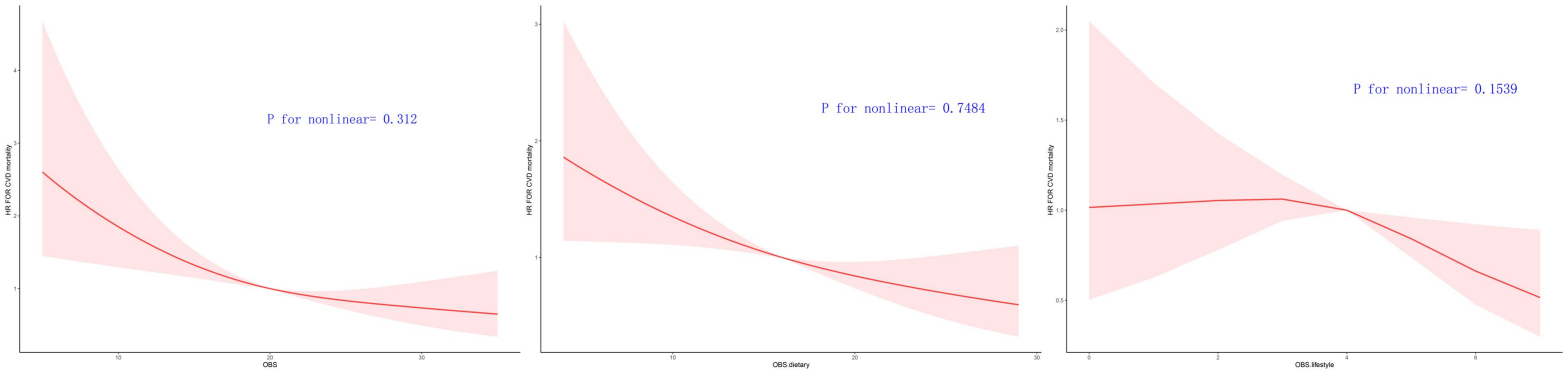
Multivariable-adjusted HRs for CVD mortality by OBS, diatery OBS, and lifestyle OBS.

Figures 3, 4 illustrate the correlation between OBS, dietary OBS, lifestyle OBS, and both all-cause mortality and CVD mortality.

After accounting for Model 3, a linear correlation was observed between OBS, dietary OBS, and the mortality rates for all causes and cardiovascular diseases, as indicated in Table 3 and Supplementary Table S3. According to RCS models, the aHRs for mortality from any cause and cardiovascular disease exhibited a consistent decline as OBS and dietary OBS increase, as shown in Figures 3, 4.

A linear correlation was found between lifestyle OBS and CVD mortality in the OBS for lifestyle. The RCS models indicated that the aHRs for CVD mortality exhibit a consistent decline as lifestyle OBS increases (Figure 4.). However, the RCS models indicated a turning point for overall mortality at 4 (Figures 3, 4). On the left side of this inflection point, the HR for all-cause mortality was 1.000 [95% CI: (0.910, 1.099), *p* = 0.996]. The HR for all-cause mortality on the right side of the inflection point was 0.836 [(95% CI 0.661, 1.057), *p* = 0.134] in Figure 3.

Supplementary Figures S4–S9 show the trend of decreasing HR for all-cause and CVD mortality with increasing OBS/dietary OBS/lifestyle OBS, which remained in male subgroups. However dietary OBS (*p* overall = 0.096) and lifestyle OBS (*p* overall = 0.6271) was not found to reduce CVD mortality in the female group (*p* overall = 0.096) (Supplementary Figures S7, S9).

**Figure 5:**
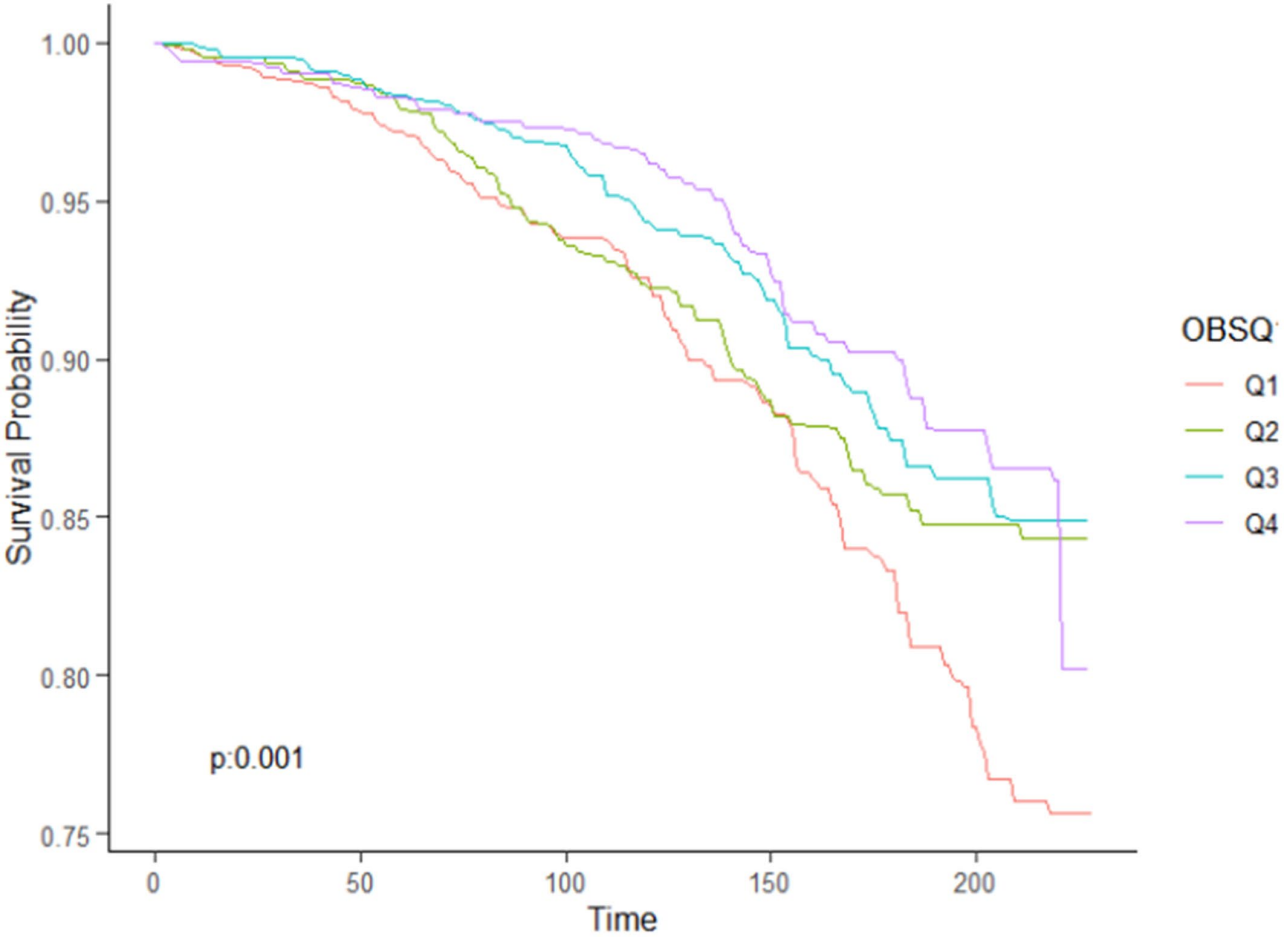
Kaplan–Meier survival curves for all cause mortality.

As depicted in the Kaplan–Meier survival curves, patients with high ASCVD risk who had OBS < 15 (Q1) exhibited significantly higher rates of all-cause mortality (Figure 5) during the follow-up period.

## Discussion

The current research revealed that elevated OBS was linked to a reduced 10-year ASCVD risk and exhibited a negative relationship between OBS and all-cause, CVD mortality in individuals with high ASCVD risk. The significance of a diet and lifestyle rich in antioxidants is underscored by our research, particularly in terms of reducing the 10-year ASCVD risk, all-cause and CVD mortality with high ASCVD risk. As far as we know, this research is the first to assess the correlation between OBS and all-cause mortality, as well as CVD mortality in individuals with a high ASCVD risk.

The current research shows a negative correlation between OBS and the 10-year ASCVD risk. The lifestyle and dietary factors of OBS have a significant impact on CVD (23). Previous studies have established that harmful lifestyles, such as obesity, excessive alcohol intake, and lack of physical activity can influence the development of CVD (24–26). According to the Global Burden of Disease Study (27), approximately 52% of all CVD deaths worldwide were attributed to risks associated with diet. The development of CVD and CVD mortality is widely recognized to be influenced significantly by chronic inflammation and oxidative stress (28). Studies conducted earlier have suggested that the overall eating pattern and different elements of the diet are directly linked to inflammation (29–32). Different dietary elements have also been linked to playing a significant part in the progression of different CVD (33–35). A meta-analysis of 14 studies investigated the link between the Dietary Inflammatory Index (DII) and CVD revealed compelling evidence indicating a significant association between a higher inflammatory diet and increased risk of CVD and related mortality (36). Oxidative stress refers to the in equilibrium between substances that promote oxidation and those that inhibit oxidation. The composite dietary antioxidant index (CDAI), is a score that assesses the overall antioxidant capacity of a person’s diet. It takes into account different vitamins and minerals that have antioxidant properties, such as vitamins A, C, and E, as well as selenium and zinc (37, 38). A cross-sectional study discovered that multiple factors indicating a reduced occurrence of ASCVD in postmenopausal females exhibiting elevated CDAI levels, as well as a dose–response relationship in the L shape between CDAI levels and the hazards of ASCVD (16). Insufficient evidence exists to support the cardioprotective advantages of vitamin and mineral supplementation. A study in an elderly Swedish population discovered that providing selenium and coenzyme Q10 supplements to a group of elderly individuals who had low levels of selenium and coenzyme Q10 resulted in a decreased risk of CVD mortality when compared to the placebo group (39). Nevertheless, in the previous year, the US Preventive Services Task Force (USPSTF) stirred controversy when they performed a comprehensive evaluation of the effectiveness and possible drawbacks of a single nutrient, nutrient pair, or multivitamin supplementation in adults to lower the chances of developing CVD, cancer, and mortality (40). To prevent CVD (41), the USPSTF recommendation goes against specific vitamin supplements, like beta-carotene and vitamin E, which were previously believed to possess antioxidant properties, according to their findings. In a recent update of the previous systematic review and meta-analysis conducted in 2018, it was found that commonly used multivitamins, vitamin D, calcium, and vitamin C had no impact on cardiovascular disease outcomes and all-cause mortality, however, an elevated risk of all-cause mortality was observed when niacin was taken with statin medication (42). It is important to mention that nutrients are not ingested in solitude but rather as a component of a food matrix. Hence, it proves challenging to regulate the potential impacts of additional nutrients offered by a food source. In recent times, the emphasis of nutritional studies on cardiovascular disease has changed from individual nutrients and particular foods to overall eating habits (43). Examining entire dietary patterns may hold greater significance and be more easily understood when compared to analyzing individual nutrients or specific food items. This could be partly due to the fact that numerous connections between specific elements of macronutrients and the risk of CVD are not linear, resulting in perplexing and contradictory results that do not align with present dietary guidelines. Additional research on chronic illnesses has similarly discovered that the collective influence of various elements may have a greater correlation with the risk of developing diseases when compared to the impact of individual nutrients (44, 45). In this study, we opted for OBS as a measure of the overall equilibrium between pro-oxidants and antioxidants in our diet and lifestyle. In addition, we uncovered the significance of OBS in predicting the 10-year ASCVD risk. We believe that incorporating a composite measure of oxidative balance, which includes a combination of dietary patterns and lifestyle factors related to pro-oxidant and antioxidant exposures, may have a stronger association with the 10-year ASCVD risk.

Inadequate nutrition is a prominent contributing factor to all-cause mortality, CVD mortality, and cancer mortality (46). The quality of dietary intake and its correlation with the risk of mortality showed significant variations among European nations. In Spain, the Mediterranean diet showed a significant negative correlation with mortality, whereas in the Netherlands, the Healthy Nordic Dietary Pattern was found to be strongly linked to reduced mortality. These results reflect dietary cultural and pattern differences. A comprehensive analysis of multiple studies indicated that following a diet rich in inflammatory components could potentially increase the chances of developing colorectal cancer, CVD, and all-cause mortality (47). A Pan-European Cohort Study has revealed that different measures of diet quality are linked to all-cause mortality, as well as cause-specific (CVD and cancer) mortality, especially with stronger associations with CVD compared to cancer, and these scores have poor predictive performance for 10-year mortality risk when used in isolation. However, in combination with other non-invasive common risk factors such as smoking, body weight, physical activity, and educational level, these composite scores display good predictive ability (23). These studies support a view whereby it is necessary to analyze various dietary components and lifestyle as an organic whole, due to the inseparable characteristics. In our research, we have discovered a direct correlation between OBS, dietary OBS, and lifestyle OBS and the all-causes and CVD mortality. However, we have observed a non-linear relationship between lifestyle OBS and mortality rates from all causes in individuals with a high ASCVD risk, as determined by Cox proportional hazards models. According to the RCS models, there was a consistent decline in the aHRs for all-causes and CVD mortality as the OBS, dietary OBS, and lifestyle OBS increased. In the female group, the subgroup analysis indicated that there was no reduction in CVD mortality with the use of OBS in the diet or lifestyle (Supplementary Figures S6–S9). We infer that it may be hormonally related. The antioxidant effects of diet and lifestyle on CVD mortality may be overshadowed by the protective function of estrogen, resulting in a lack of statistical significance in the correlation.

## Strengths and limitations

### Strengths

The present study had multiple advantages. First, the rare exploration of OBSs potential protection against ASCVD was not commonly undertaken in previous studies. Second, the extensive size of the sample and rigorous statistical analyses conducted on the overall population allowed us to extend our findings to the majority of individuals. Third, to ensure the accuracy of the results, this study controlled many confounding factors, including creatinine, WBC, ALT, alcohol user, hypertension, hyperlipidemia, and anemia. In our study, we initially performed cross-sectional analyses followed by additional cohort analyses.

### Limitations

In addition, there are various drawbacks in the present investigation. Incorporating all ASCVD-associated dietary and lifestyle exposures proved challenging for the OBS; the database had restrictions on various elements, including flavonoids. Moreover, there was a possibility that certain ambiguous ASCVD-related dietary or lifestyle factors were not encompassed. Furthermore, the OBS dietary constituents were obtained from self-reported information collected through a single 24 h, which could introduce measurement inaccuracies and biases, and might not accurately reflect day-to-day variations in diet, thereby resulting in imprecise estimations. Lastly, it was not possible to utilize any biomarkers to authenticate the accuracy of the OBS in evaluating oxidative balance in the current investigation. Nevertheless, the association between 10-year ASCVD and the current OBS remained consistent and is unlikely to be significantly influenced by the excluded elements.

## Conclusion

The findings of this research demonstrate that individuals with elevated OBS levels exhibited a decreased likelihood of developing ASCVD risk over a span of 10 years. Moreover, it suggests that adopting a dietary pattern and lifestyle that promotes higher OBS levels can be advantageous in mitigating the risk of mortality associated with all-cause and CVD among individuals with a high predicted 10-year ASCVD risk.

## Data availability statement

The original contributions presented in the study are included in the article/Supplementary material, further inquiries can be directed to the corresponding authors.

## Ethics statement

The studies involving humans were approved by the NCHS Research Ethics Review Board at the National Center for Health Statistics. The studies were conducted in accordance with the local legislation and institutional requirements. Written informed consent for participation was not required from the participants or the participants’ legal guardians/next of kin in accordance with the national legislation and institutional requirements.

## Author contributions

DJ: Writing – original draft. TL: Data curation, Methodology, Software, Writing – original draft. SC: Methodology, Writing – original draft. YC: Methodology, Writing – original draft. CZ: Conceptualization, Supervision, Writing – review & editing. XW: Conceptualization, Supervision, Writing – review & editing. JL: Conceptualization, Supervision, Writing – review & editing.

## Supplementary material

SUPPLEMENTARY FIGURE S1 Linear association between OBS and 10-years ASCVD risk by sex.

SUPPLEMENTARY FIGURE S2 Linear association between dietary OBS and 10-years ASCVD risk by sex.

SUPPLEMENTARY FIGURE S3 Linear association between lifestyle OBS and 10-years ASCVD risk by sex.

SUPPLEMENTARY FIGURE S4 Linear association between OBS and all cause mortality by sex.

SUPPLEMENTARY FIGURE S5 Linear association between OBS and CVD mortality by sex.

SUPPLEMENTARY FIGURE S6 Linear association between dietary OBS and all cause mortality by sex.

SUPPLEMENTARY FIGURE S7 Linear association between diatery OBS and CVD mortality by sex.

SUPPLEMENTARY FIGURE S8 Linear association between lifestyle OBS and all cause mortality by sex.

SUPPLEMENTARY FIGURE S9 Linear association between lifestyle OBS and CVD mortality by sex.
