# Supplementary material for: Association between oxidative balance score and 10-year atherosclerotic cardiovascular disease risk: results from the NHANES database
Source: Front Nutr. 2024 Jul 15;11:1422946. doi: 10.3389/fnut.2024.1422946 (PMC11284129; doi:10.3389/fnut.2024.1422946)
Supplement: Supplementary file 1 [file Data_Sheet_1.zip › Supplementary Table 1.docx]

|  | Multivariable adjusted (OR, 95% CI)* | | | | | |
| --- | --- | --- | --- | --- | --- | --- |
|  | Model 1 |  | Model 2 |  | Model 3 |  |
| Dietary OBS | 95%CI | P | 95%CI | P | 95%CI | P |
| Q1[1,11] | ref |  | ref |  | ref |  |
| Q2(11,17] | 0.89(0.76,1.04) | 0.13 | 0.93( 0.75, 1.16) | 0.52 | 0.87( 0.67, 1.14) | <0.0001 |
| Q3(11,17] | 0.77(0.66,0.90) | 0.001 | 0.67( 0.53, 0.85) | <0.001 | 0.67( 0.49, 0.93) | <0.0001 |
| Q4 (22,31] | 0.54(0.46,0.63) | <0.0001 | 0.47( 0.37, 0.60) | <0.0001 | 0.49( 0.33, 0.72) | <0.0001 |
| P trend |  | <0.0001 |  | <0.0001 |  | <0.0001 |
| Dietary OBS | 0.96(0.96,0.97) | <0.0001 | 0.96( 0.94, 0.97) | <0.0001 | 0.96( 0.93, 0.98) | <0.0001 |
| Lifestyle OBS | 95%CI | P | 95%CI | P | 95%CI | P |
| Q1[0,3] | ref |  | ref |  | ref |  |
| Q2 (3,4] | 0.87(0.75,1.00) | 0.05 | 0.43( 0.34, 0.54) | <0.0001 | 0.49( 0.36, 0.65) | 0.31 |
| Q3(4,5] | 0.89(0.78,1.02) | 0.10 | 0.32( 0.26, 0.40) | <0.0001 | 0.44( 0.35, 0.55) | 0.02 |
| Q4(5,7] | 0.69(0.59,0.80) | <0.0001 | 0.18( 0.14, 0.23) | <0.0001 | 0.34( 0.26, 0.45) | <0.001 |
| P trend |  | <0.0001 |  | <0.0001 |  | <0.001 |
| Lifestyle OBS | 0.92(0.89,0.95) | <0.0001 | 0.64( 0.60, 0.68) | <0.0001 | 0.74( 0.69, 0.79) | <0.0001 |

**Table S1.** Multivariable Logistic regression analyses demonstrating associations of dietary OBS, lifestyle OBS and 10-year ASCVD risk. Model 1 comprised OBS lifestyle and OBS dietary. Model 2 included Model 1, age, sex, race, education and marital. Model 3 included Model 2, creatinine, lymphocyte ratio(LYM), leucocyte count(WBC), glutamic-pyruvic transaminase(ALT), alcohol user, diabetes mellitus(DM), hypertension, hyperlipidemia, anemia, total energy intake. *P<0.05.
