# Supplementary material for: Association between oxidative balance score and 10-year atherosclerotic cardiovascular disease risk: results from the NHANES database
Source: Front Nutr. 2024 Jul 15;11:1422946. doi: 10.3389/fnut.2024.1422946 (PMC11284129; doi:10.3389/fnut.2024.1422946)
Supplement: Supplementary file 1 [file Data_Sheet_1.zip › Supplementary Table 2.docx]

|  | Multivariable adjusted (OR, 95% CI)* | | | |
| --- | --- | --- | --- | --- |
|  |  | OR(95% CI) | p | p for interaction |
| OBS |  |  |  |  |
|  | Sex |  |  | 0.445 |
|  | Female | 0.942(0.920, 0.966) | <0.0001 |  |
|  | Male | 0.939(0.914, 0.964) | <0.0001 |  |
|  | Aged |  |  | 0.088 |
|  | <60 | 0.945(0.924, 0.966) | <0.0001 |  |
|  | >60 | 0.975(0.948, 1.003) | 0.084 |  |
|  | GFR |  |  | 0.234 |
|  | 60-90 | 0.935( 0.909, 0.962) | <0.0001 |  |
|  | >=90 | 0.945( 0.918, 0.971) | <0.001 |  |
|  | <60 | 0.895( 0.827, 0.969) | 0.007 |  |
|  | Hyperlipidemia |  |  | 0.005 |
|  | yes | 0.938( 0.918, 0.958) | <0.0001 |  |
|  | no | 0.941( 0.887, 0.998) | 0.043 |  |
|  | Hypertension |  |  | 0.13 |
|  | no | 0.934( 0.911, 0.957) | <0.0001 |  |
|  | yes | 0.943( 0.917, 0.970) | <0.0001 |  |
| lifestyle OBS |  |  |  |  |
|  | sex |  |  | 0.216 |
|  | Female | 0.747(0.680, 0.820) | <0.0001 |  |
|  | Male | 0.736(0.674, 0.803) | <0.0001 |  |
|  | aged |  |  | 0.001 |
|  | <60 | 0.834( 0.779, 0.893) | <0.0001 |  |
|  | >60 | 0.926( 0.847, 1.012) | 0.088 |  |
|  | GFR |  |  | 0.004 |
|  | 60-90 | 0.727( 0.659, 0.802) | <0.0001 |  |
|  | >=90 | 0.718( 0.647, 0.797) | <0.0001 |  |
|  | <60 | 0.872( 0.649, 1.171) | 0.359 |  |
|  | Hyperlipidemia |  |  | < 0.0001 |
|  | yes | 0.725( 0.681, 0.772) | <0.0001 |  |
|  | no | 0.851( 0.710, 1.019) | 0.079 |  |
|  | Hypertension |  |  | 0.39 |
|  | no | 0.699( 0.637, 0.766) | <0.0001 |  |
|  | yes | 0.759( 0.688, 0.837) | <0.0001 |  |
|  |  |  |  |  |
| dietary OBS |  |  |  |  |
|  | sex |  |  | 0.649 |
|  | Female | 0.957(0.931, 0.985) | 0.003 |  |
|  | Male | 0.953(0.926, 0.981) | 0.002 |  |
|  | aged |  |  | 0.269 |
|  | <60 | 0.951(0.928, 0.975) | <0.001 |  |
|  | >60 | 0.978(0.948, 1.009) | 0.164 |  |
|  | GFR |  |  | 0.47 |
|  | 60-90 | 0.950( 0.920, 0.980) | 0.002 |  |
|  | >=90 | 0.963( 0.935, 0.992) | 0.012 |  |
|  | <60 | 0.893( 0.820, 0.972) | 0.01 |  |
|  | Hyperlipidemia |  |  | 0.062 |
|  | yes | 0.954( 0.932, 0.977) | <0.001 |  |
|  | no | 0.943( 0.884, 1.006) | 0.077 |  |
|  | Hypertension |  |  | 0.14 |
|  | no | 0.951( 0.926, 0.977) | <0.001 |  |
|  | yes | 0.957( 0.928, 0.986) | 0.005 |  |

**Table S2.** Multivariable Logistic regression analyses demonstrating associations of OBS, diatery OBS, lifestyle OBS and 10-year ASCVD risk by sex,aged,GFR,Hyperlipidemia and Hypertension. *P<0.05.
