# Supplementary material for: Association between oxidative balance score and 10-year atherosclerotic cardiovascular disease risk: results from the NHANES database
Source: Front Nutr. 2024 Jul 15;11:1422946. doi: 10.3389/fnut.2024.1422946 (PMC11284129; doi:10.3389/fnut.2024.1422946)
Supplement: Supplementary file 1 [file Data_Sheet_1.zip › Supplementary Table 3.docx]

|  | Toatl | Q1 | Q2 | Q3 | Q4 | Pvalue |
| --- | --- | --- | --- | --- | --- | --- |
| age | 64.018(0.173) | 62.985(0.297) | 63.694(0.349) | 63.912(0.347) | 65.284(0.396) | < 0.001 |
| sex |  |  |  |  |  | 0.377 |
| Female | 1670(33.482) | 372(30.250) | 457(34.731) | 397(33.550) | 444(34.804) |  |
| Male | 3222(66.518) | 904(69.750) | 857(65.269) | 721(66.450) | 740(65.196) |  |
| edu |  |  |  |  |  | < 0.0001 |
| <high | 1265(15.196) | 446(22.050) | 370(15.523) | 260(14.559) | 189( 9.859) |  |
| college | 2421(59.418) | 473(46.892) | 604(57.112) | 592(60.701) | 752(70.773) |  |
| high | 1206(25.386) | 357(31.058) | 340(27.365) | 266(24.739) | 243(19.368) |  |
| eth1 |  |  |  |  |  | < 0.0001 |
| black | 969( 8.359) | 376(14.218) | 282( 9.347) | 153( 5.512) | 158( 5.189) |  |
| other | 1406(12.809) | 361(15.298) | 380(11.378) | 326(12.899) | 339(12.132) |  |
| white | 2517(78.832) | 539(70.484) | 652(79.276) | 639(81.589) | 687(82.679) |  |
| marital |  |  |  |  |  | 0.04 |
| marry | 3062(66.499) | 748(61.569) | 831(67.577) | 704(67.340) | 779(68.670) |  |
| non-marry | 1830(33.501) | 528(38.431) | 483(32.423) | 414(32.660) | 405(31.330) |  |
| Alcohol user |  |  |  |  |  | < 0.0001 |
| former | 1185(20.652) | 359(26.493) | 328(19.410) | 249(18.823) | 249(18.802) |  |
| heavy | 648(13.731) | 180(14.884) | 193(16.588) | 158(15.016) | 117( 8.753) |  |
| mild | 1926(43.551) | 420(33.339) | 495(41.762) | 462(45.328) | 549(52.050) |  |
| moderate | 537(11.838) | 162(14.117) | 137(11.736) | 125(11.558) | 113(10.335) |  |
| never | 596(10.229) | 155(11.167) | 161(10.504) | 124( 9.274) | 156(10.060) |  |
| total energy intake (kcal) | 2115.920(19.242) | 1578.133(31.258) | 1935.418(26.364) | 2272.358(42.241) | 2592.745(37.537) | < 0.0001 |
| Creatinine(mg.dl) | 0.962(0.006) | 1.010(0.014) | 0.966(0.011) | 0.948(0.008) | 0.933(0.008) | < 0.0001 |
| Wbc(1000cells.ul) | 7.279(0.050) | 7.597(0.088) | 7.342(0.078) | 7.404(0.130) | 6.840(0.080) | < 0.0001 |
| Lym(%) | 28.959(0.143) | 29.128(0.327) | 28.722(0.290) | 29.085(0.353) | 28.943(0.295) | 0.845 |
| Hemoglobin(g/dl) | 14.700(0.039) | 14.748(0.074) | 14.700(0.065) | 14.724(0.055) | 14.638(0.063) | 0.62 |
| Alt(U/L) | 26.190(0.355) | 26.275(0.675) | 27.380(1.037) | 25.789(0.565) | 25.295(0.499) | 0.283 |
| dietary OBS |  |  |  |  |  |  |
| carotene_RE | 221.512(6.397) | 117.114( 9.581) | 165.357( 9.145) | 220.777(11.852) | 363.622(15.820) | < 0.0001 |
| dietary_fiber(g) | 17.575(0.177) | 9.946(0.141) | 14.671(0.228) | 18.510(0.256) | 25.857(0.420) | < 0.0001 |
| total_fat(g) | 80.920(0.797) | 58.045(1.117) | 74.436(1.200) | 88.378(1.629) | 99.272(1.480) | < 0.0001 |
| alpha_carotene_mcg | 452.558(19.487) | 251.411(23.885) | 358.114(28.562) | 430.494(34.429) | 731.412(53.120) | < 0.0001 |
| beta_carotene_mcg | 2431.862(69.460) | 1279.657(106.476) | 1805.222(100.020) | 2434.082(128.718) | 3997.756(169.664) | < 0.0001 |
| riboflavin_mg | 2.269(0.026) | 1.449(0.021) | 1.946(0.022) | 2.447(0.034) | 3.099(0.060) | < 0.0001 |
| niacin_mg | 25.024(0.290) | 16.376(0.268) | 21.900(0.340) | 27.110(0.421) | 33.305(0.560) | < 0.0001 |
| vitamin_B6_mg | 2.073(0.027) | 1.206(0.021) | 1.724(0.030) | 2.242(0.045) | 2.974(0.053) | < 0.0001 |
| total_folate_mcg | 413.577(4.518) | 239.906( 3.251) | 341.073( 5.183) | 438.999( 6.418) | 604.709(10.001) | < 0.0001 |
| vitamin_B12_mcg | 5.642(0.203) | 2.975(0.088) | 4.251(0.103) | 6.253(0.294) | 8.655(0.614) | < 0.0001 |
| vitamin_C_mg | 86.752(1.665) | 45.266(2.122) | 70.153(2.302) | 93.139(4.418) | 131.399(2.966) | < 0.0001 |
| vitamin_E_ATE_mg | 8.064(0.112) | 4.560(0.088) | 6.583(0.117) | 8.544(0.161) | 11.969(0.236) | < 0.0001 |
| calcium_mg | 910.594(10.384) | 549.499(10.863) | 773.518(13.018) | 976.921(16.015) | 1281.993(22.164) | < 0.0001 |
| magnesium_mg | 304.398(2.696) | 185.978(2.475) | 255.933(2.340) | 331.722(3.688) | 424.635(5.545) | < 0.0001 |
| iron_mg | 15.683(0.185) | 9.697(0.170) | 13.372(0.199) | 16.481(0.264) | 22.154(0.366) | < 0.0001 |
| zinc_mg | 12.070(0.176) | 7.468(0.145) | 9.978(0.177) | 13.203(0.380) | 16.884(0.326) | < 0.0001 |
| copper_mg | 1.362(0.030) | 0.805(0.012) | 1.098(0.014) | 1.460(0.036) | 1.992(0.088) | < 0.0001 |
| selenium_mcg | 112.744(0.939) | 75.287(1.223) | 100.626(1.323) | 121.533(1.518) | 147.422(2.426) | < 0.0001 |
| OBS.dietary | 16.400(0.154) | 7.656(0.097) | 13.548(0.084) | 18.833(0.073) | 24.171(0.095) | < 0.0001 |
| lifestyle OBS |  |  |  |  |  |  |
| Alcohol(g) | 10.379(0.618) | 9.961(0.865) | 9.476(0.990) | 12.982(1.514) | 9.252(0.699) | 0.106 |
| BMI(kg/m^2^) | 29.133(0.118) | 29.851(0.235) | 29.533(0.229) | 29.344(0.230) | 27.953(0.192) | < 0.0001 |
| Physical activity | 2890.120(91.007) | 2450.964(141.428) | 2735.574(143.664) | 3016.849(205.099) | 3287.594(191.363) | 0.002 |
| cotinine_ng/ml | 68.019(3.337) | 118.102(8.812) | 68.292(5.926) | 55.670(4.717) | 38.170(4.850) | < 0.0001 |
| smoke |  |  |  |  |  | < 0.0001 |
| No | 3745(76.019) | 857(63.014) | 995(75.195) | 861(78.016) | 1032(85.629) |  |
| Yes | 1147(23.981) | 419(36.986) | 319(24.805) | 257(21.984) | 152(14.371) |  |
| OBS.lifestyle | 4.140(0.035) | 3.384(0.057) | 4.051(0.061) | 4.142(0.053) | 4.845(0.054) | < 0.0001 |
| anemia |  |  |  |  |  | 0.508 |
| Mild | 301( 4.010) | 93(5.218) | 83(3.831) | 62(3.691) | 63(3.495) |  |
| Moderate | 42( 0.608) | 16(1.101) | 14(0.625) | 6(0.444) | 6(0.337) |  |
| Non-Anaemia | 4548(95.376) | 1167(93.681) | 1217(95.543) | 1049(95.841) | 1115(96.167) |  |
| Severe | 1( 0.006) | 0(0.000) | 0(0.000) | 1(0.024) | 0(0.000) |  |
| Hyperlipidemia |  |  |  |  |  | 0.033 |
| no | 730(13.131) | 185(11.754) | 189(11.660) | 156(12.301) | 200(16.486) |  |
| yes | 4162(86.869) | 1091(88.246) | 1125(88.340) | 962(87.699) | 984(83.514) |  |
| CKD |  |  |  |  |  | 0.036 |
| no | 3716(76.748) | 916(72.153) | 1006(76.691) | 873(78.533) | 921(78.924) |  |
| yes | 1176(23.252) | 360(27.847) | 308(23.309) | 245(21.467) | 263(21.076) |  |
| DM |  |  |  |  |  | 0.138 |
| no | 3337(73.070) | 849(72.397) | 898(71.524) | 760(71.643) | 830(76.470) |  |
| yes | 1555(26.930) | 427(27.603) | 416(28.476) | 358(28.357) | 354(23.530) |  |
| Hypertension |  |  |  |  |  | 0.069 |
| no | 1623(34.575) | 378(32.098) | 452(33.665) | 374(32.843) | 419(39.088) |  |
| yes | 3269(65.425) | 898(67.902) | 862(66.335) | 744(67.157) | 765(60.912) |  |
| 10-years ASCVD risk | 0.182(0.002) | 0.193(0.004) | 0.181(0.004) | 0.183(0.004) | 0.173(0.004) | 0.005 |
| status |  |  |  |  |  | < 0.0001 |
| alive | 3850(81.268) | 934(75.240) | 1049(81.851) | 872(80.745) | 995(86.075) |  |
| death | 1042(18.732) | 342(24.760) | 265(18.149) | 246(19.255) | 189(13.925) |  |

Table S3: Basic characteristics of the cohort study.
