# Supplementary material for: Association between oxidative balance score and 10-year atherosclerotic cardiovascular disease risk: results from the NHANES database
Source: Front Nutr. 2024 Jul 15;11:1422946. doi: 10.3389/fnut.2024.1422946 (PMC11284129; doi:10.3389/fnut.2024.1422946)
Supplement: Supplementary file 1 [file Data_Sheet_1.zip › Supplementary Table 4.docx]

|  | Multivariable adjusted (HR, 95% CI)* | | | | | |
| --- | --- | --- | --- | --- | --- | --- |
|  | Model 1 | | Model 2 | | Model 3 | |
|  | 95%CI | P | 95%CI | P | 95%CI | P |
| all-cause mortality |  |  |  |  |  |  |
| Dietary OBS | 0.97(0.96,0.99) | <0.0001 | 0.97(0.96,0.98) | <0.0001 | 0.98(0.96,0.99) | 0.002 |
| Lifestyle OBS | 1.02(0.98,1.07) | 0.38 | 0.89(0.85,0.93) | <0.0001 | 0.94(0.89,0.99) | 0.01 |
| CVD mortality |  |  |  |  |  |  |
| Dietary OBS | 0.96(0.94,0.98) | <0.001 | 0.96(0.93,0.98) | <0.001 | 0.95(0.93,0.98) | 0.002 |
| Lifestyle OBS | 1.02(0.94,1.10) | 0.69 | 0.84(0.77,0.91) | <0.0001 | 0.90(0.82,0.98) | 0.02 |

**Table S4.** HR for associations between OBS dietary, OBS lifestyle and all-cause mortality, CVD mortality. Model 1 comprised OBS dietary and OBS lifestyle. Model 2 comprised Model 1, age and sex. Model 3 comprised Model 2, race, eduation, creatinine, lymphocyte ratio(LYM), leucocyte count(WBC), glutamic-pyruvic transaminase(ALT), alcohol user, diabetes mellitus(DM), hypertension, hyperlipidemia, anemia, total energy intake. *P<0.05.
