# Supplementary figures and images for: Association between oxidative balance score and 10-year atherosclerotic cardiovascular disease risk: results from the NHANES database
Source: Front Nutr. 2024 Jul 15;11:1422946. doi: 10.3389/fnut.2024.1422946 (PMC11284129; doi:10.3389/fnut.2024.1422946)

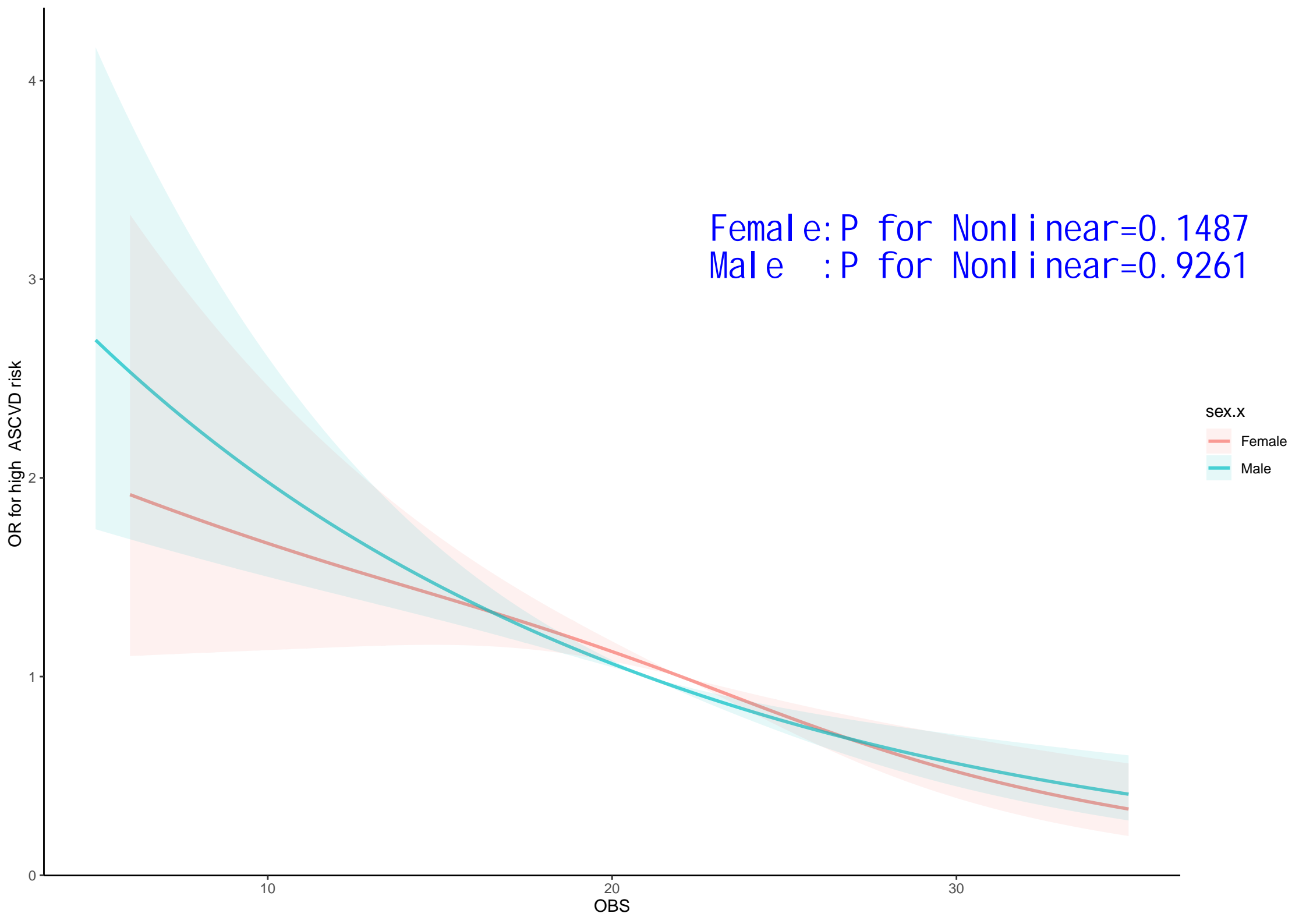

Supplement: Supplementary file 1 [file Data_Sheet_1.zip › Supplementary Image 1.PDF]

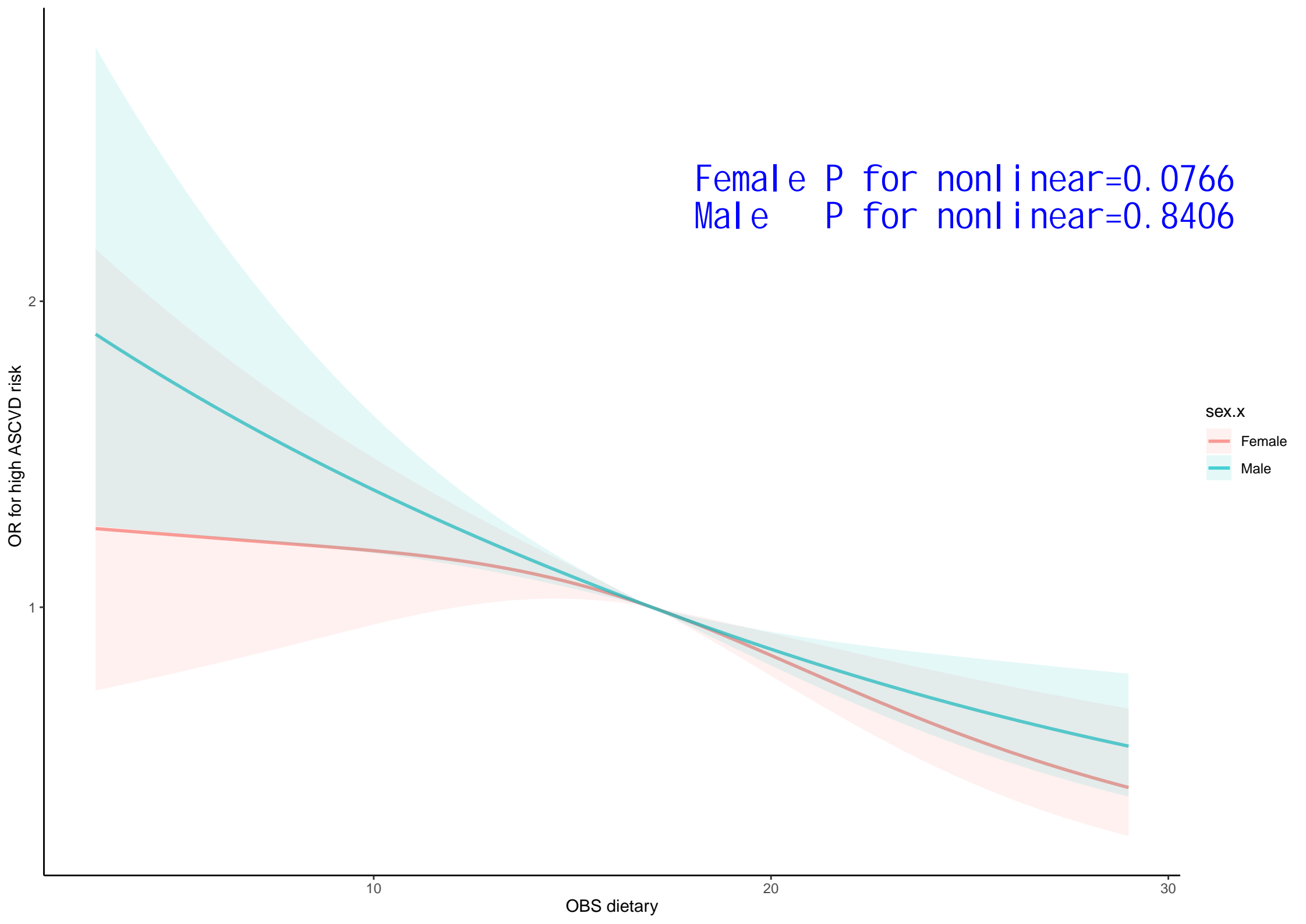

Supplement: Supplementary file 1 [file Data_Sheet_1.zip › Supplementary Image 2.PDF]

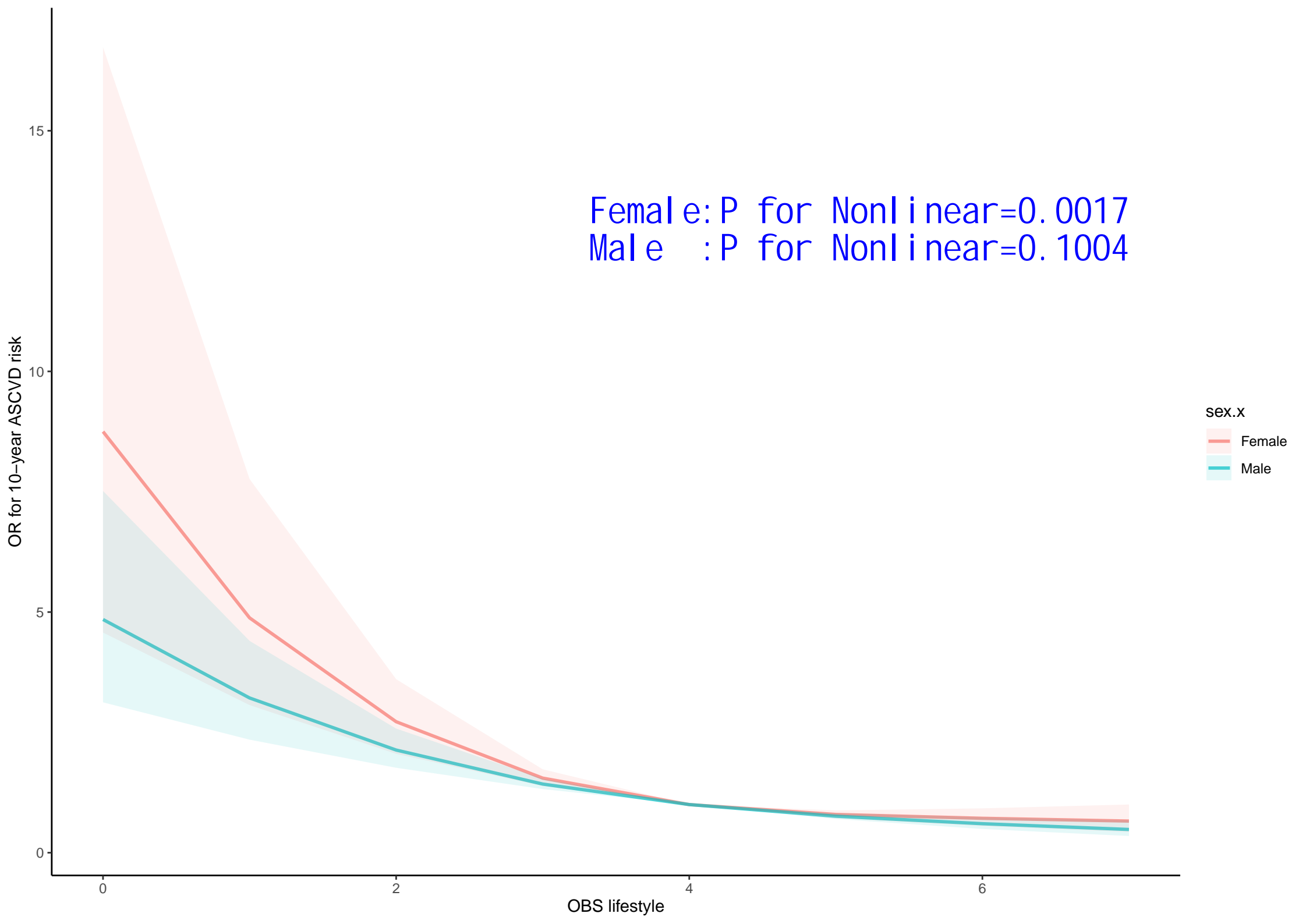

Supplement: Supplementary file 1 [file Data_Sheet_1.zip › Supplementary Image 3.PDF]

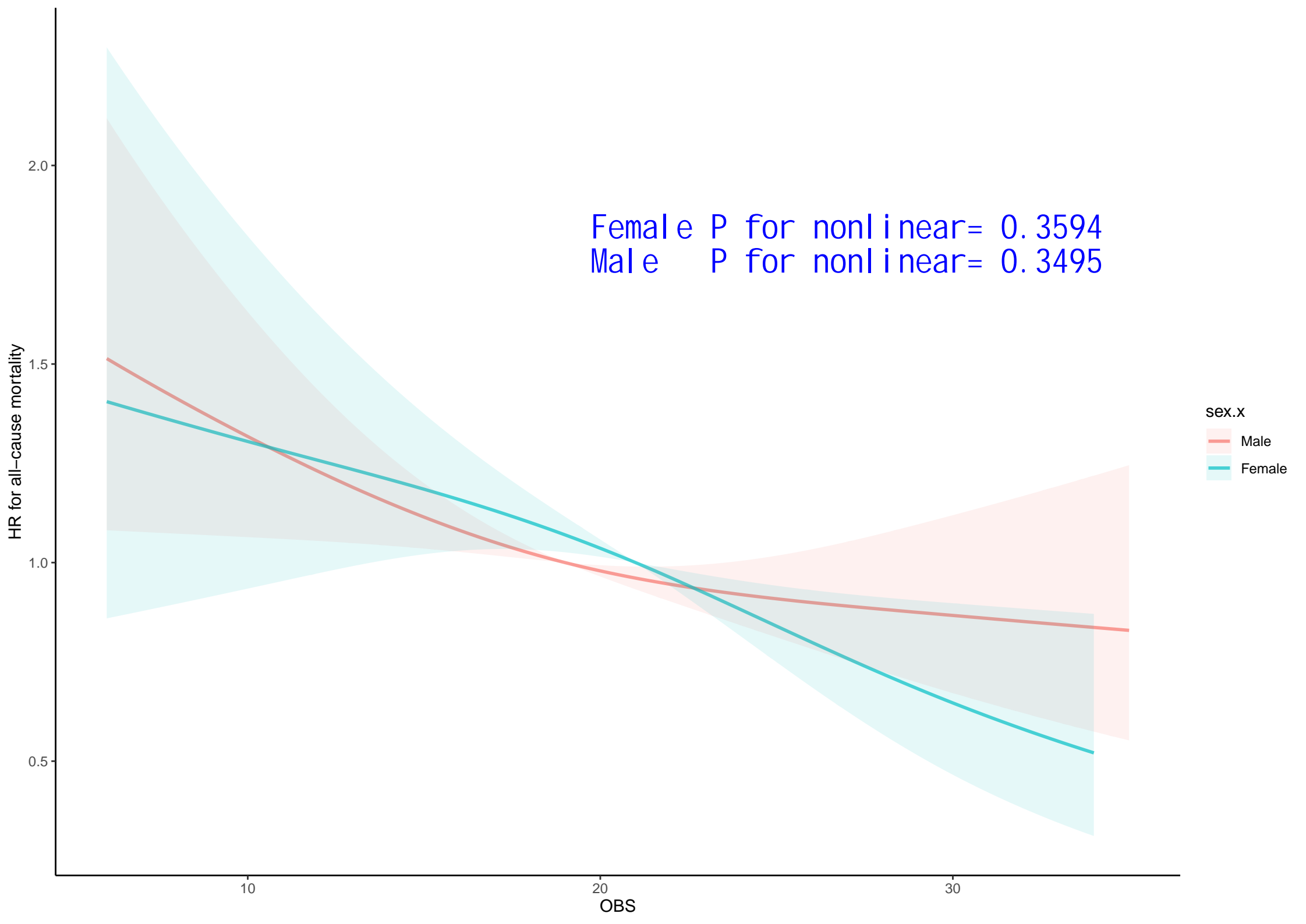

Supplement: Supplementary file 1 [file Data_Sheet_1.zip › Supplementary Image 4.PDF]

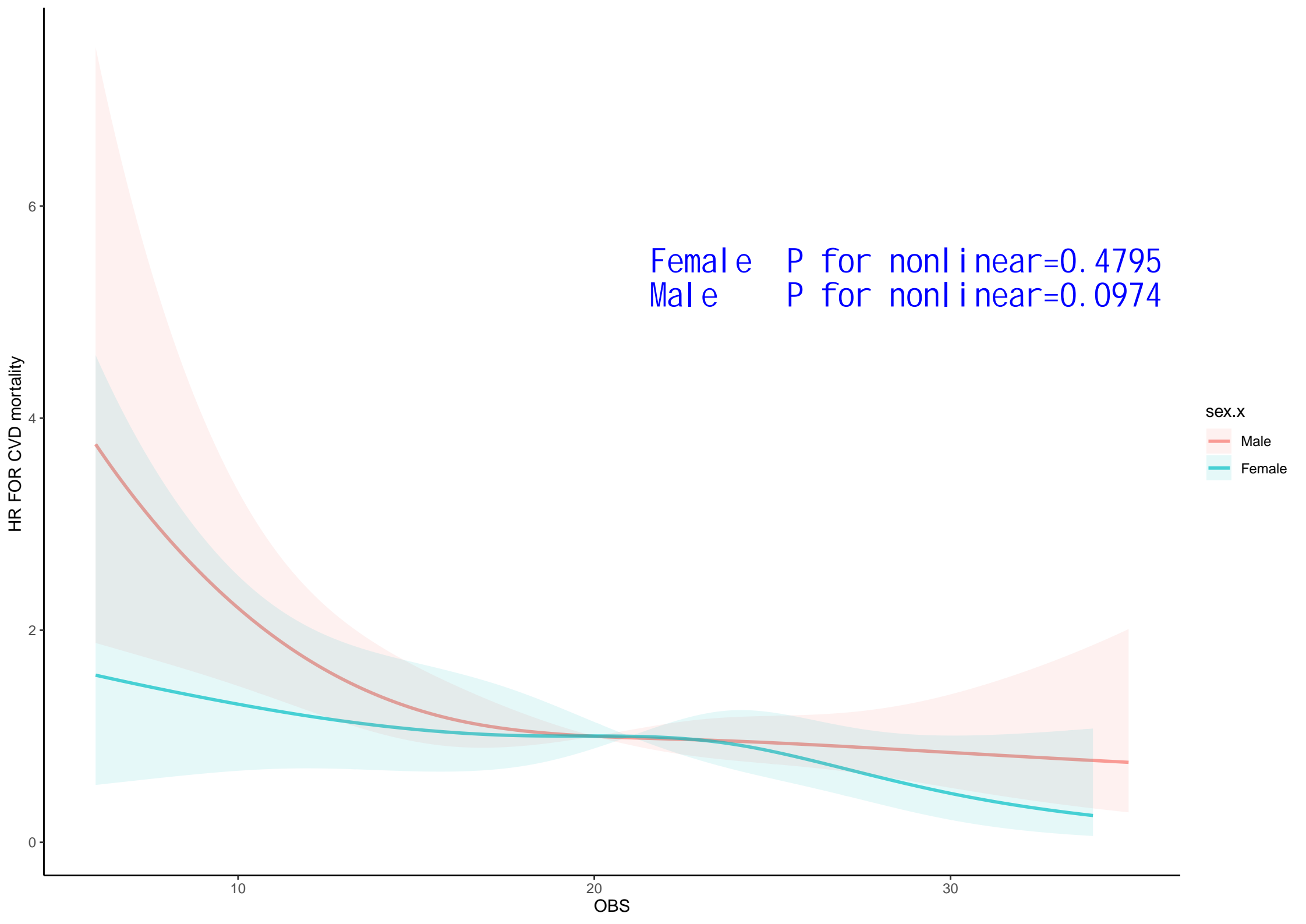

Supplement: Supplementary file 1 [file Data_Sheet_1.zip › Supplementary Image 5.PDF]

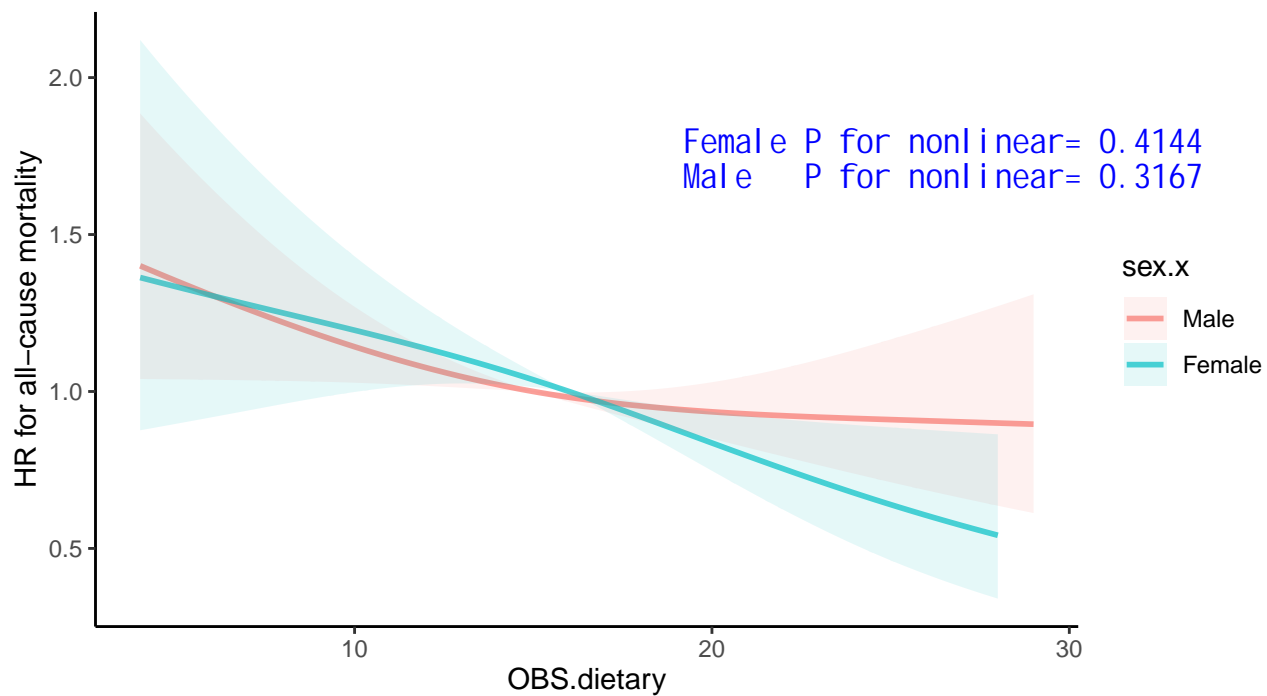

Supplement: Supplementary file 1 [file Data_Sheet_1.zip › Supplementary Image 6.PDF]

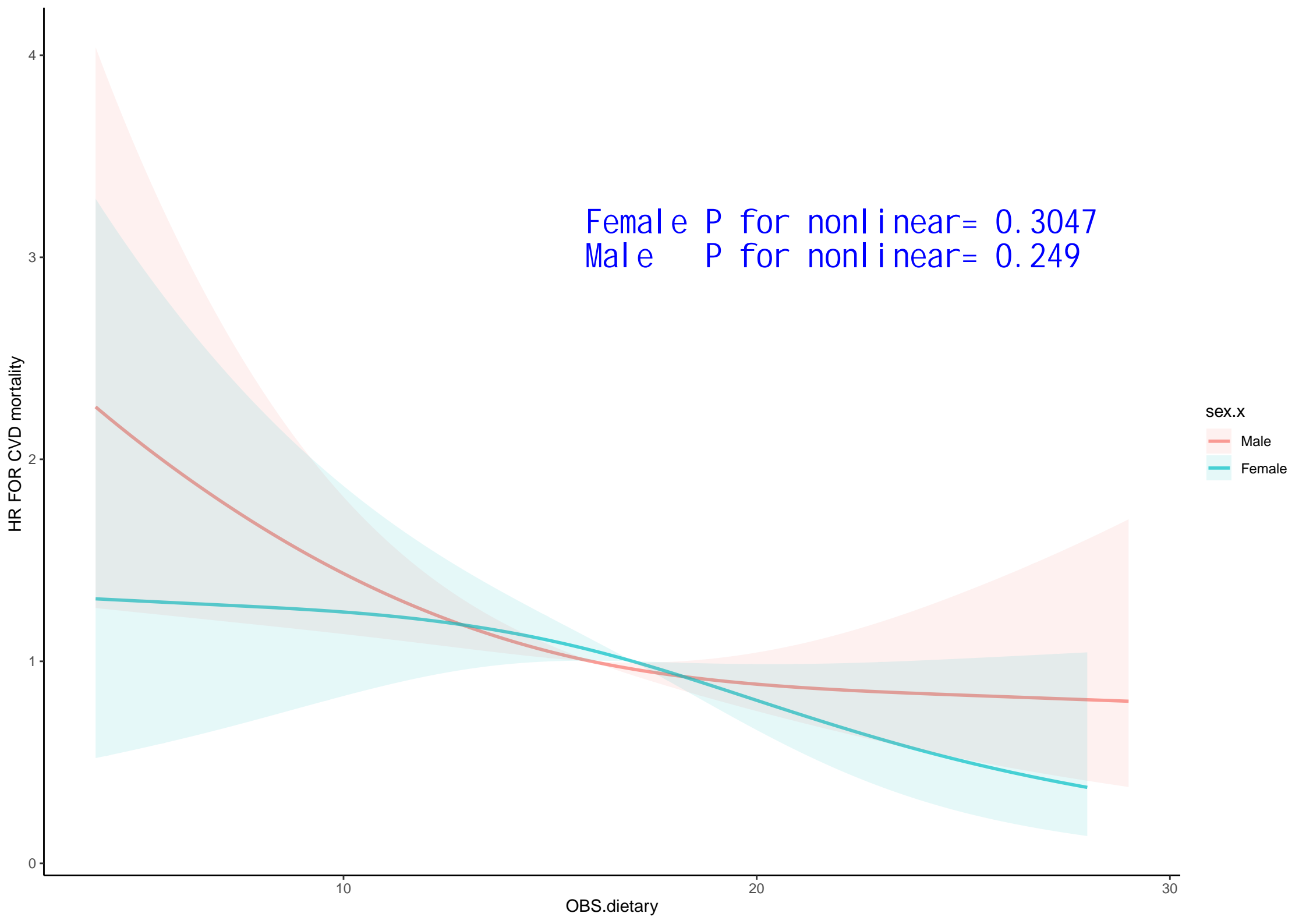

Supplement: Supplementary file 1 [file Data_Sheet_1.zip › Supplementary Image 7.PDF]

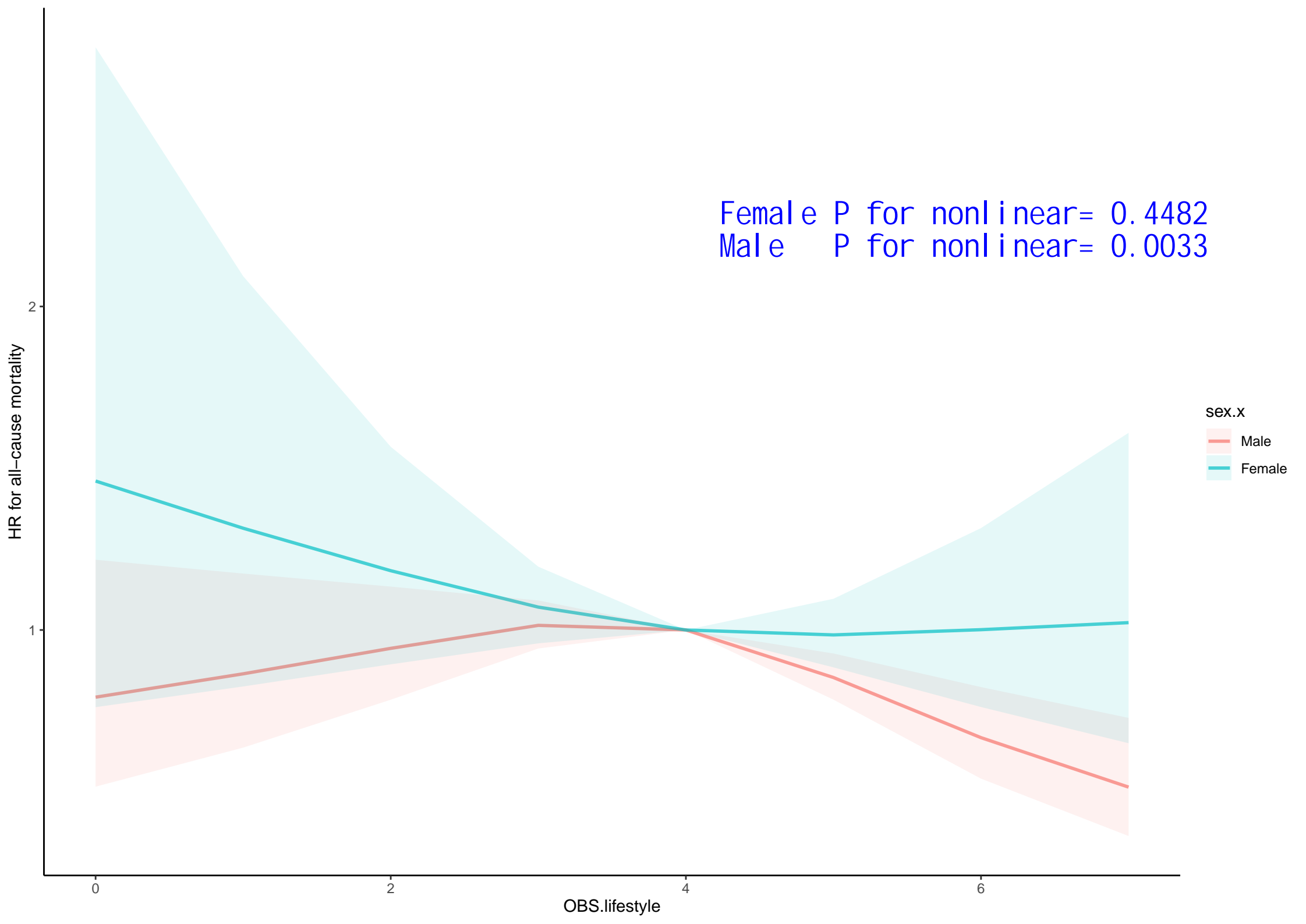

Supplement: Supplementary file 1 [file Data_Sheet_1.zip › Supplementary Image 8.PDF]

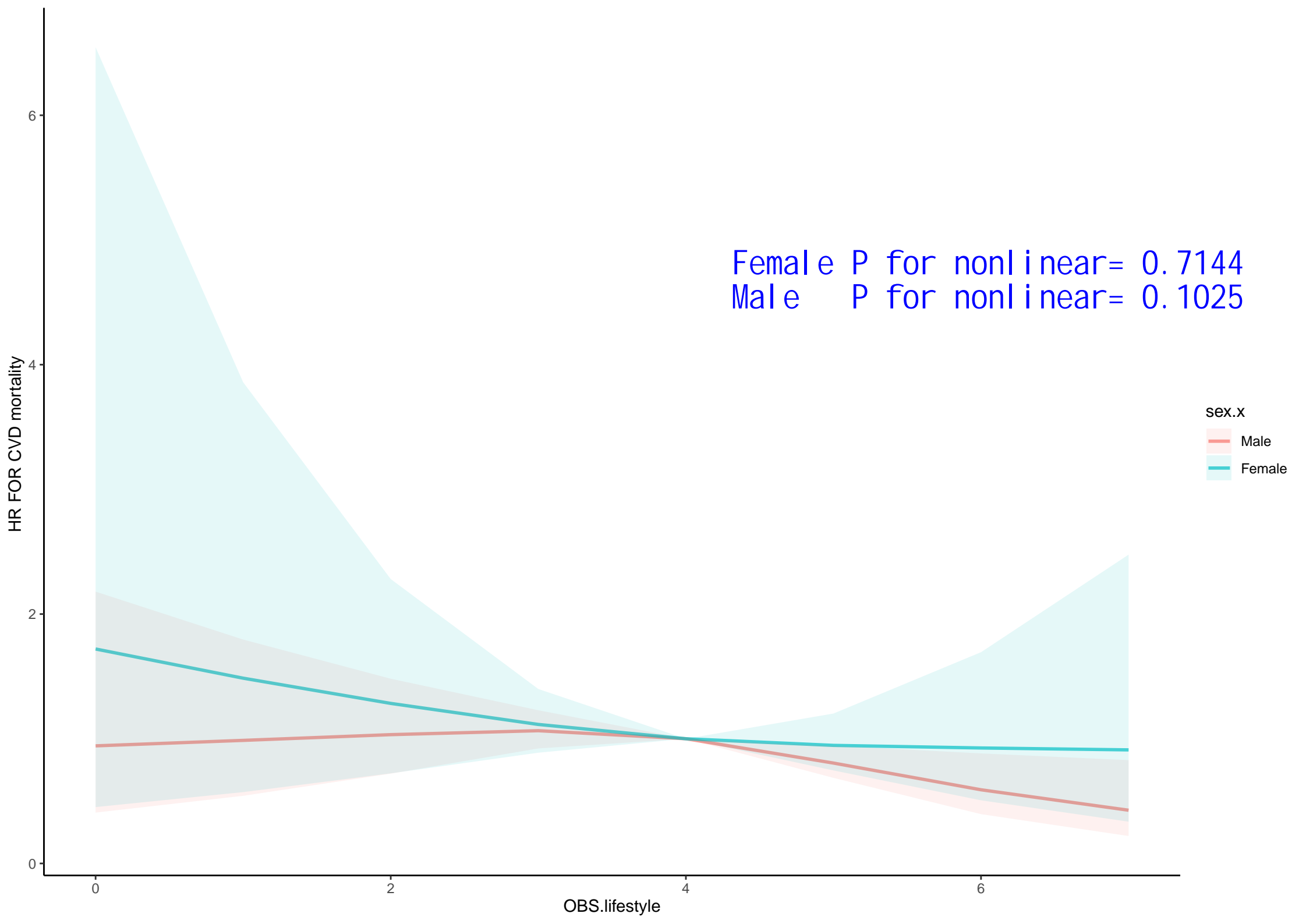

Supplement: Supplementary file 1 [file Data_Sheet_1.zip › Supplementary Image 9.PDF]
